# Microbial Sulfide Filter along a Benthic Redox Gradient in the Eastern Gotland Basin, Baltic Sea

**DOI:** 10.3389/fmicb.2017.00169

**Published:** 2017-02-09

**Authors:** Mustafa Yücel, Stefan Sommer, Andrew W. Dale, Olaf Pfannkuche

**Affiliations:** ^1^GEOMAR Helmholtz Centre for Ocean Research KielKiel, Germany; ^2^Middle East Technical University, Institute of Marine SciencesErdemli, Turkey

**Keywords:** voltammetry, redox, sediments, sulfur, baltic sea, gotland basin, beggiatoa, sulfur oxidizers

## Abstract

The sediment-water interface is an important site for material exchange in marine systems and harbor unique microbial habitats. The flux of nutrients, metals, and greenhouse gases at this interface may be severely dampened by the activity of microorganisms and abiotic redox processes, leading to the “benthic filter” concept. In this study, we investigate the spatial variability, mechanisms and quantitative importance of a microbially-dominated benthic filter for dissolved sulfide in the Eastern Gotland Basin (Baltic Sea) that is located along a dynamic redox gradient between 65 and 173 m water depth. In August-September 2013, high resolution (0.25 mm minimum) vertical microprofiles of redox-sensitive species were measured in surface sediments with solid-state gold-amalgam voltammetric microelectrodes. The highest sulfide consumption (2.73–3.38 mmol m^−2^ day^−1^) occurred within the top 5 mm in sediments beneath a pelagic hypoxic transition zone (HTZ, 80–120 m water depth) covered by conspicuous white bacterial mats of genus *Beggiatoa*. A distinct voltammetric signal for polysulfides, a transient sulfur oxidation intermediate, was consistently observed within the mats. In sediments under anoxic waters (>140 m depth), signals for Fe(II) and aqueous FeS appeared below a subsurface maximum in dissolved sulfide, indicating a Fe(II) flux originating from older sediments presumably deposited during the freshwater Ancylus Lake that preceded the modern Baltic Sea. Our results point to a dynamic benthic sulfur cycling in Gotland Basin where benthic sulfide accumulation is moderated by microbial sulfide oxidation at the sediment surface and FeS precipitation in deeper sediment layers. Upscaling our fluxes to the Baltic Proper; we find that up to 70% of the sulfide flux (2281 kton yr^−1^) toward the sediment-seawater interface in the entire basin can be consumed at the microbial mats under the HTZ (80–120 m water depth) while only about 30% the sulfide flux effuses to the bottom waters (>120 m depth). This newly described benthic filter for the Gotland Basin must play a major role in limiting the accumulation of sulfide in and around the deep basins of the Baltic Sea.

## Introduction

Hydrogen sulfide accumulation in marine sediments results from sulfate reduction, which is a globally important organic carbon oxidation pathway (Jørgensen and Kasten, 2006). Only a small fraction of the sulfide produced escapes to water column due to the abundantly available O_2_ in the bottom waters and surface oxidized sediment layer. The flux of sulfide to bottom waters becomes more likely under low oxygen (hypoxic) to oxygen-free (anoxic) bottom waters, the extent of which have been increasing due to eutrophication in coastal areas (Diaz and Rosenberg, 2008). Even under low-O_2_ conditions, the upward fluxes of sulfide, potentially toxic to pelagic organisms, may still be dampened due to microbial sulfide oxidation and abiotic processes such as metal oxide reduction (Poulton et al., 2004). These biotic and abiotic processes can occur over such small scales (mm) that the uppermost section of the sediments may be viewed as a benthic filter, with significant consequences for the overlying water column ecosystem.

Seafloor microbial mats of sulfide-oxidizing bacteria are important in the modulation of benthic fluxes (Revsbech and Jørgensen, 1983). In order to quantify the processes occurring within these mats, high-resolution (sub mm) gradients need to be detected as sulfide is cycled very close to the sediment-water interface over spatial scales typically smaller than traditional sampling resolutions involving porewater extraction (0.5–1 cm). One approach to overcome this shortcoming is to obtain higher resolution vertical profiles using electrochemical microsensors (Taillefert et al., 2000; Kühl and Revsbech, 2001). Such profiles, mostly obtained by amperometric sulfide sensors (Kühl and Revsbech, 2001) revealed that mm-scale steep sulfide gradients close the sediment-water interface result in high sulfide fluxes (typically larger than 1 mmol m^−2^ day^−1^). These fluxes, when appropriate electron acceptors are available, can support mats of sulfide-oxidizing bacteria in shallow water marine sediments (Preisler et al., 2007), anoxic basins (Jessen et al., 2016), sediments beneath upwelling areas (Ferdelman et al., 1997), cold seeps (de Beer et al., 2006), and hydrothermal vents (Wenzhöfer et al., 2000). However, a stronger case for sulfide oxidation pathways and products can only be established if various sulfide oxidation intermediates are also documented at the same time, such as FeS, polysulfides (${\text{S}}_{\text{x}}^{2-}$) and thiosulfate (S_2_${\text{O}}_{3}^{-2}$). Among electrochemical microsensors, voltammetric microsensors used in this study are particularly tailored toward sulfur oxidation studies as they are simultaneously sensitive to hydrogen sulfide, polysulfides and FeS.

Accurate quantification of benthic sulfide fluxes is particularly important for anoxic marine systems, in which euxinic conditions persist in the water column either permanently or episodically. In the modern ocean, such areas include landlocked environments such as the Black Sea and Cariaco Basin as well as intense ocean margin oxygen minimum zones (Lavik et al., 2009; Schunck et al., 2013; Sommer et al., 2016). The deeper part of the Eastern Gotland Basin (EGB) in the Baltic Sea is also such an environment. The pelagic redoxline of the EGB hosts sulfur redox processes where metal cycling (Neretin et al., 2003) coupled to sulfide oxidation by Mn(IV), Fe(III) as well as ${\text{NO}}_{3}^{-}$ yields local concentration maxima of zerovalent sulfur (S_8_) and thiosulfate (S_2_${\text{O}}_{3}^{-2}$; Kamyshny et al., 2013). The depth distribution of these products can be variable due to seasonal physical and biogeochemical forcings (Meyer et al., 2014). In contrast to the water column, benthic sulfur cycling in the EGB has received relatively less attention with the majority of sediment geochemistry studies aiming at reconstruction of past environmental changes, (Sohlenius et al., 1996; Sternbeck and Sohlenius, 1997; Boettcher and Lepland, 2000) and to coupled metal-nutrient cycling over the Holocene (Heiser et al., 2001; Jilbert and Slomp, 2013a,b; Scholz et al., 2013; Lenz et al., 2014). One significant recent finding has been the first observation of extensive mats of sulfide-oxidizing bacteria covering large areas of seafloor beneath a water column HTZ in the EGB (Noffke et al., 2016). This hints at a widespread yet unquantified benthic sulfide filter operating in this fragile, anthropogenically-impacted environment where seafloor elemental cycling may provide important feedback to water column biogeochemical processes.

Here we investigate benthic sulfur redox cycling in the upper sediments of the EGB using high vertical resolution (min. 0.25 mm) chemical profiles obtained with multi-analyte sensitive voltammetric microelectrodes. We supplement these microprofiles with sulfide fluxes obtained with benthic lander deployments and profiles of particulate and porewater constituents measured on vertical core sections. With these datasets, we aim to (i) resolve the vertical gradients and fluxes of electroactive porewater sulfur-iron species along a benthic redox gradient and (ii) estimate relative magnitudes of dissolved sulfide flux to the water column and sulfide retention within sediments under the hypoxic and anoxic bottom waters of the basin.

## Materials and methods

### Study area

The Baltic Sea is a shallow, landlocked brackish marine environment and contains several deep basins where a stable water column halocline limits exchanges between upper and deeper water layers (Matthaeus, 1995). Since the last deglaciation ca 13,000 BP, the environment transformed from an ice-dammed lake to a marine environment for a brief period (Yoldia Sea, 10,000–9500 BP), followed by another lacustrine period (Ancylus Lake - Sohlenius et al., 1996; Sternbeck and Sohlenius, 1997). Since ca 8000 BP, the marine connection was established again through the Danish straits. This Littorina Sea period has been characterized by basin-wide intermittent hypoxia (Zillen et al., 2008; Jilbert and Slomp, 2013a). The deep basins were probably continuously anoxic throughout the entirety of the Littorina period as shown from laminated sediment sequences obtained from the EGB (max. depth 250 m) and Landsort Deep (max. depth 450 m). However, the EGB in particular is not permanently anoxic due to the episodic introduction of oxygenated waters originating from North Sea that leads to a temporary period (weeks–months) of deep-water oxygenation (Matthaeus, 1995). Most recent ventilation of the EGB occurred during March–December 2015 (Mohrholz et al., 2015). Prior to this, stagnant euxinic conditions prevailed for about a decade—the last major ventilation event was in 2003 (Mohrholz et al., 2015). Therefore, our results represent the final stage of the stagnant conditions (see Noffke et al., 2016 for details on water column biogeochemistry at our study sites before prior to ventilation, and see Sommer et al., 2017 for conditions after the ventilation).

### Sediment sampling and geochemical measurements

**Figure 1:**
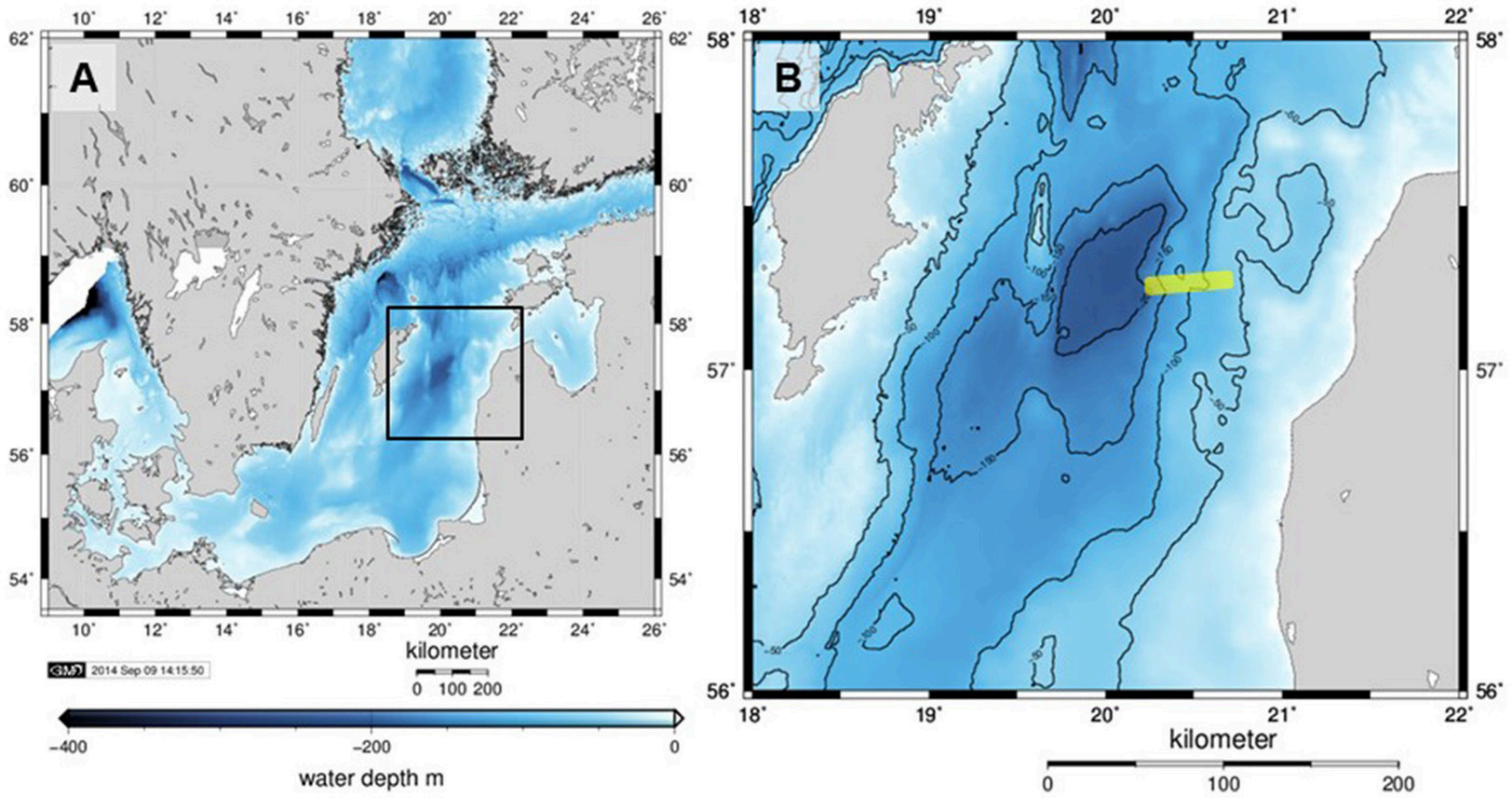
**(A)** Bathymetry (m below mean sea level) of the Baltic Proper with the Eastern Gotland Basin indicated by the black rectangle and shown in detail in **(B)**. The sampled transect was on the eastern slope of the basin and its approximate location is shown as a yellow line. Axes labels denote eastings and northings. The maps were generated with Generic Mapping Tools.

Sediment cores were obtained via a video-guided multiple-corer (MUC) during Alkor cruise 422 to the EGB between August 15 and September 15, 2013 (Figure 1). Typically, a sediment column 40–50 cm long and 10 cm wide was retrieved in fine-grained muddy sediment. Shorter cores were obtained where the sediment was hard clay (see Results). Different cores were used for microprofiling and sectioning. The latter were immediately transferred to a cool room (10°C) and sectioned under an argon atmosphere. Porewater was extracted by centrifugation. The core sections were used for the determination of particulate organic carbon and sulfur (POC, PS, Carlo Erba elemental analyzer), porewater sulfate (${\text{SO}}_{4}^{2-}$, ion chromatography), total dissolved sulfide (ΣH_2_S, colorimetry/methylene blue) and dissolved iron (Fe(II), colorimetry/ferrozine). Details on analytical methods are given in Dale et al. (2013). At certain sites, shorter sediment cores (max. 20 cm long) were taken from the benthic chamber of autonomous biogeochemical observatory lander system (BIGO; Sommer et al., 2010). A total of 8 stations between 65 and 173 m water depths were sampled (Table 1). The bottom waters of the shallowest station (65 m) were oxygenated. Four stations (82, 95, 110, and 122 m) were located under the waters of a hypoxic transition zone (HTZ, here broadly defined as the zone between 80 and 120 m, [O_2_] < 30 μM, [H_2_S] < 1 μM) while the “deep basin” corresponds to area beyond 120 m depth where three of our stations (140, 152, and 173 m) were located (see the companion paper Noffke et al., 2016 for the water column redox state terminology). The deep basin of the EGB is characterized by higher concentrations of reducing chemical species such as H_2_S and ammonium (${\text{NH}}_{4}^{+}$).

**Table 1:** **Details of sediment sampling during Alkor cruise ALK 422 to Gotland Basin**.

**Depth, m**	**Gear**	**ALK 422 station**	**Date (2013)**	**Lat. °N**	**Lon. °E**	**Bottom water [O_2_], [H_2_S] in μM**	**Visible mats**
65	MUC12	653	9.9	57°26.52′	20°43.55′	>300, 0	no
65	BIGO2-6	651	10.9	57°26.26′	20°43.53′	>300, 0	yes
82	MUC5	581	23.8	57°21.81′	20°35.88′	10, 0	yes
95	MUC1	559	19.8	57°20.76′	20°35.30′	<5, 0	yes
95	MUC9	614	4.9	57°20.76′	20°35.32′	bdl	yes
95	BIGO2-1	561	21.8	57°20.76′	20°35.32′	bdl	yes
110	MUC7	596	26.8	57°20.58′	20°34.33′	bdl	yes
110	BIGO2-3	600	28.8	57°20.58′	20°34.32′	bdl	yes
123	MUC2	564	20.8	57°18.51′	20°33.00′	bdl, 9	yes
123	BIGO1-1	568	22.8	57°18.51′	20°32.99′	bdl, 9	yes
140	BIGO2-5	635	8.9	57°14.99′	20°27.13′	bdl, 46	no
152	MUC8	602	27.8	57°20.95′	20°28.99′	bdl, 77	no
152	BIGO1-3	603	29.8	57°20.98′	20°28.99′	bdl, 77	no
173	MUC6	587	24.8	57°21.05′	20°27.95′	bdl, 153	no
173	BIGO2-4	618	6.9	57°21.05′	20°27.97′	bdl, 173	no

“*MUC” refers to a multicore sediment sample and BIGO stands for the “Biogeochemical Observatory” benthic lander. The first number following the BIGO operations indicates the lander used (#1 or #2) and the second to the deployment number. The redox conditions of the bottom water and the visible presence of surface bacterial mats are also given. (bdl, below detection limit)*.

### Voltammetric microsensor measurements and diffusive flux calculations

We measured the electroactive redox chemical species in the sediment porewaters with a three-electrode voltammetric sensor with the gold amalgam (Au/Hg) voltammetric glass microelectrode as the working electrode (Luther et al., 1998). These electrodes can simultaneously measure porewater redox species such as O_2_, HS^−^/H_2_S, S_2_${\text{O}}_{3}^{2-}$, ${\text{S}}_{\text{x}}^{2-}$, S^0^, Mn^2+^, Fe^2+^, and qualitatively detect soluble FeS_aq_ and Fe^3+^ (Brendel and Luther, 1995; Taillefert et al., 2007; Luther et al., 2008). The three-electrode cell was calibrated separately for O_2_, Mn^2+^, Fe^2+^, and H_2_S before the first application using standard additions. Mn^2+^ was used as a pilot ion for subsequent calibrations (Konovalov et al., 2007; Slowey and Marvin-Dipasquale, 2012; Yücel, 2013). Data were recorded from the electrodes using a bench-top potentiostat (DLK-60, AIS, Inc.).

The retrieved sediment cores were profiled with the voltammetric microelectrode within 1 h of core retrieval. Replicate profiles were obtained on different spots on the same core. The Au/Hg glass working electrode was attached to a micromanipulator (Maerzhaeuser MM33) with counter and reference electrodes placed in the overlying water of the core. The working electrode was then vertically maneuvered with a minimum step of 0.25 mm. Four voltammetric scans were taken at each depth increment. The scans were taken in cyclic voltammetry form, starting from −0.1 V to −1.8 V and back at a rate of 1000 mV s^−1^. Before each scan the electrode was electrochemically conditioned at −0.9 V for 10 s to remove any adsorbed species (Konovalov et al., 2007; Yücel, 2013). The concentrations were calculated using triplicate measurements after discarding the first scan, which yielded standard deviations <5%. Detection limits (DL) were 20 μM for O_2_, 10 μM for Mn^2+^ and Fe^2+^ and 0.2 μM for H_2_S. Calibration of FeS_aq_ is not possible due to lack of standards and only the signal intensity will be reported here. For sulfide, cyclic voltammetry gives two signals. On the one hand, a sharp peak proportional to the concentration of HS^−^/H_2_S can be detected at ca. −0.7 V while scanning forward (toward the negative voltages). This peak shifts to more negative values (−0.9 V) with increasing sulfide concentrations. FeS_aq_ is also detectable at −1.1 V (Theberge and Luther, 1997; Bura-Nakic et al., 2007). On the other hand, these sulfide species give a single wave-like signal during the backward scan (−1.8 V to −0.1 V) that is proportional to the sum of dissolved sulfide species (HS^−^, H_2_S, ${\text{S}}_{\text{x}}^{2-}$, and labile metal sulfides such as FeS). This is defined here as total dissolved sulfide concentration (ΣH_2_S) since it represents a larger dynamic range (2–1200 μM). The forward signal is more sensitive (0.2 μM detection limit) yet typically saturated around 250 μM.

Diffusive fluxes (*J*, mmol m^−2^ day^−1^) were calculated from the concentration data using Fick's First Law (Equation 1), using measured linear concentration gradients and assuming steady-state conditions:

(1) $$\begin{array}{l} J=\varphi \left(0\right)\cdot D_{S}\cdot \frac{d\text{C}\left(\text{z}\right)}{dz} \end{array}$$

where *d*C/*dz* is the linear concentration gradient (mmol m^−3^ m^−1^), φ(0) (dimensionless) is the surface sediment porosity and *D*_*S*_ (m^2^ day^−1^) is the diffusion coefficient. The latter was calculated from the diffusion coefficients in sediment-free seawater, *D* (m^2^ day^−1^), by correcting for tortuosity using the modified Weissberg relation (Boudreau, 1997):

(2) $$\begin{array}{l} D_{\text{S}}=D/\left[1-2\text{ln }\left(\varphi \left(0\right)\right)\right] \end{array}$$

Diffusion coefficients were calculated for the ambient conditions (salinity 12, T = 10°C, 1 atm) using the Stokes-Einstein relationship with R package Marelac (Soetaert et al., 2012), leading to values of 1.36 × 10^−4^, 1.02 × 10^−4^, and 0.41 × 10^−4^ m^2^ day^−1^ for O_2_, H_2_S and Fe, respectively. The concentration gradient was estimated using the LINEST function in Excel from the concentration data covering the relevant depth range (typically the top 0–5 mm). With increasing sediment depth, positive fluxes predicted by Equation (1) imply a flux directed from seawater into the sediment. In this paper, we define positive fluxes as being directed out the sediment and vice versa. The uncertainty in the fluxes was estimated as ± 2 times the standard error (95% confidence interval).

During micro-profiling, the sediment cores were open to the atmosphere and not stirred during this procedure, which raises the possibility that the O_2_ profiles (shallow site only) could have been affected by artifacts arising from (i) a change in O_2_ concentrations above the diffusive boundary layer (DBL) due to atmospheric exchange, and (ii) an increase in the thickness of the DBL due a reduction in the flow regime at the sediment surface. Quantifying these artifacts is very difficult, but replicate profiles (one after the other) were very similar (see **Figure 3D**), which suggests that any alteration of the true O_2_ gradient through the DBL may be minor. Nevertheless, we recognize the potential error in the O_2_ fluxes, but argue that the order of magnitude is correct. Besides, the O_2_ fluxes can only be treated as a snapshot of the current situation since bottom water O_2_ concentrations in the oxycline regions of the EGB are extremely variable over a 24 h period, varying by tens of μM (**Figure 3** in Noffke et al., 2016). We further add that H_2_S fluxes are much more likely to be reliable and representative of quasi-steady state conditions since they are less likely to be affected by short term changes in bottom water O_2_ concentrations.

### Benthic chamber sulfide flux measurements

Benthic flux measurements using the BIGO lander are described in detail by Sommer et al. (2010) and references therein. In short, the BIGO lander system consisted of two benthic chambers (internal diameter: 28.8 cm, area: 651.4 cm^2^). Four hours after the placement of the lander at the seafloor, the chambers were slowly (~30 cm h^−1^) driven into the sediment. The incubation lasted 36 h during which six samples were sequentially taken from the benthic chamber using glass syringes. Upon retrieval of the lander, the syringe samples were analyzed for dissolved sulfide using the same protocol as the porewater samples (Dale et al., 2014; Noffke et al., 2016). Sulfide fluxes were calculated using the slope of the time vs. concentration data and the measured sediment-free volume of the benthic chamber.

## Results

### General sedimentary properties

The changing bottom water redox conditions were reflected in the appearance of the cores. The sediment from 65 m depth underlying oxic bottom waters was light brown to brown in color at all depths. The sediments subsampled from the BIGO lander at this depth were partially covered with white bacterial mats whereas the MUC cores had no mats visible to the naked eye. Sediments in the upper 8 cm of the 82 m core were also brown to orange in color. The underlying sediment was homogeneous gray clay—a layer also present at the base of other cores (Figure S1). The sediments sampled from water depths >90 m (anoxic) were mostly fine-grained and muddy. These sediments had a distinct dark gray-brown layer in the upper 5–20 cm followed by a light gray, more homogeneous-looking depositional layer. At 140 and 152 m cores, however, this black-organic rich zone was shallower (confined to top 5 cm) compared to others. The differences in the core characteristics, also substantiated by geochemical data described below, are due to the depositional history of EGB sediments. These sediments were previously classified as “erosion, transport and sedimentation bottoms” by Jonsson et al. (1990). It is likely that cores from 140 to 152 m represent “erosion” or “transport” bottoms which are now covered by recently deposited organic-rich sediments. Therefore, here is an abrupt transition between Ancylus Lake sediments and the modern organic-rich material. In contrast, the remainder of cores represent “sedimentation” bottoms.

A side-by-side view of cores from all sampled depths is given in Figure S1. Three of seven locations had clearly-visible mats (for 140 m we used a subcore from the BIGO chamber). Table 1 lists details on all sediment samplings. For seafloor photographs of the bacterial mats, the reader is directed to Noffke et al. (2016).

### Porewater and particulate phase geochemistry

**Figure 2:**
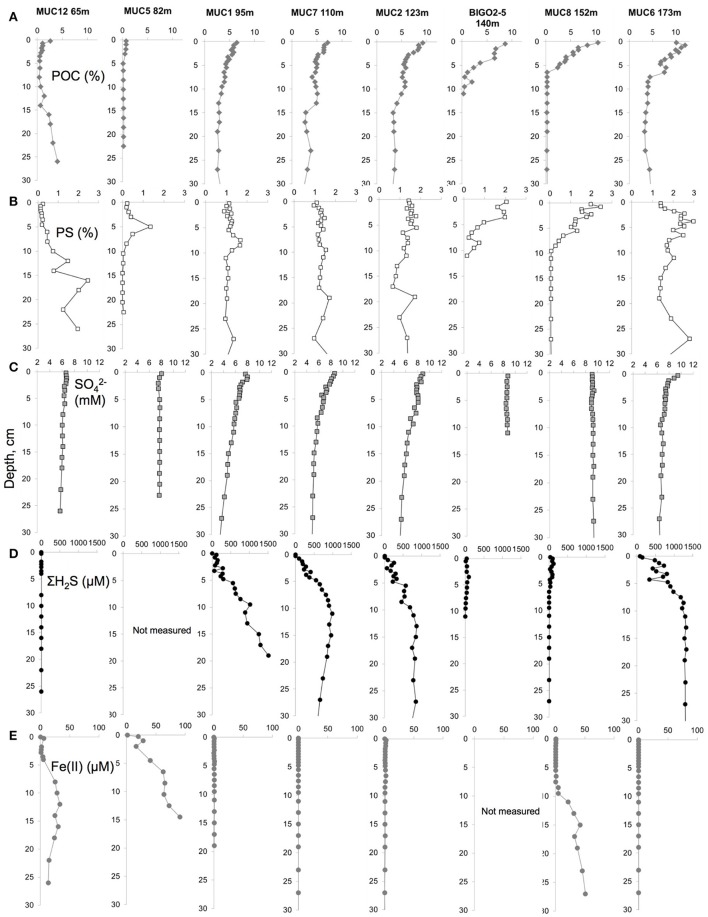
**Porewater and solid phase geochemical data for (A)** Particulate organic carbon (POC) content (%), **(B)** particulate sulfur (PS) content (%), **(C)** porewater sulfate (${\text{SO}}_{4}^{2-}$) concentration (mM), **(D)** porewater sulfide (ΣH_2_S) concentration (μM) and **(E)** porewater dissolved iron (Fe(II)) concentration (μM) in the top 30 cm of the sediments along the transect.

Concentrations of particulate and porewater constituents measured on the core sections are shown in Figure 2. A POC-rich (>3 wt.%) layer with a thickness of at least 10 cm (Figure 2A) was present in the upper part of the 95, 110, and 123 m cores. At 140 and 152 m cores the Holocene mud layer was shallower (5 cm) and the organic carbon levels decreased to below 1 wt.% in the underlying glacial clay. The surface sediments became progressively enriched in organic carbon with increasing water depth. Yet, the thickness of this mud layer was largest at the HTZ stations where the surfaces of the cores were almost completely covered by white bacterial mats and also in the deep basin. PS in the sediment solid phase also co-varied with POC, with downcore enrichment in the HTZ cores to about 1–2% and reaching highest values in the 173 m core (3%). PS contents decreased sharply within the gray-homogeneous zones at 140 and 152 m cores. As stated above, these sites represent erosional bottoms and a sharp transition from recent sediments (rich in POC/PS) to Ancylus Lake sediments (low in POC and PS).

Porewater sulfate concentrations generally decreased downcore with the strongest gradient in the cores sampled from the HTZ. For example, in the 95 m core sulfate decreased from 8 mM at the surface to 3.7 mM at 27 cm depth. The sulfate gradient appeared to track POC availability, which implies that microbial POC oxidation via sulfate reduction was the dominant remineralization process at the study sites, although a minor role for sulfate reduction coupled to the anaerobic oxidation of methane cannot be excluded (Jilbert and Slomp, 2013a; Egger et al., 2015). As a result, dissolved sulfide accumulated in the porewaters with the highest value of 1519 μM (20 cm depth) at the 95 m core. In the sediments of the 174 m core, the sulfide was high downcore but the values remained <1300 μM. Sulfide concentrations were much lower at the stations with a thin Holocene mud layer (140 and 152 m). At the highly sulfidic cores, dissolved iron was undetectable. Downcore enrichments of dissolved iron were found only in the clay layers of sediments from 65, 82, and 152 m (140 m core not measured), with a maximum of 91 μM in the 82 m core (15 cm depth).

### Voltammetric microprofiles

**Figure 3:**
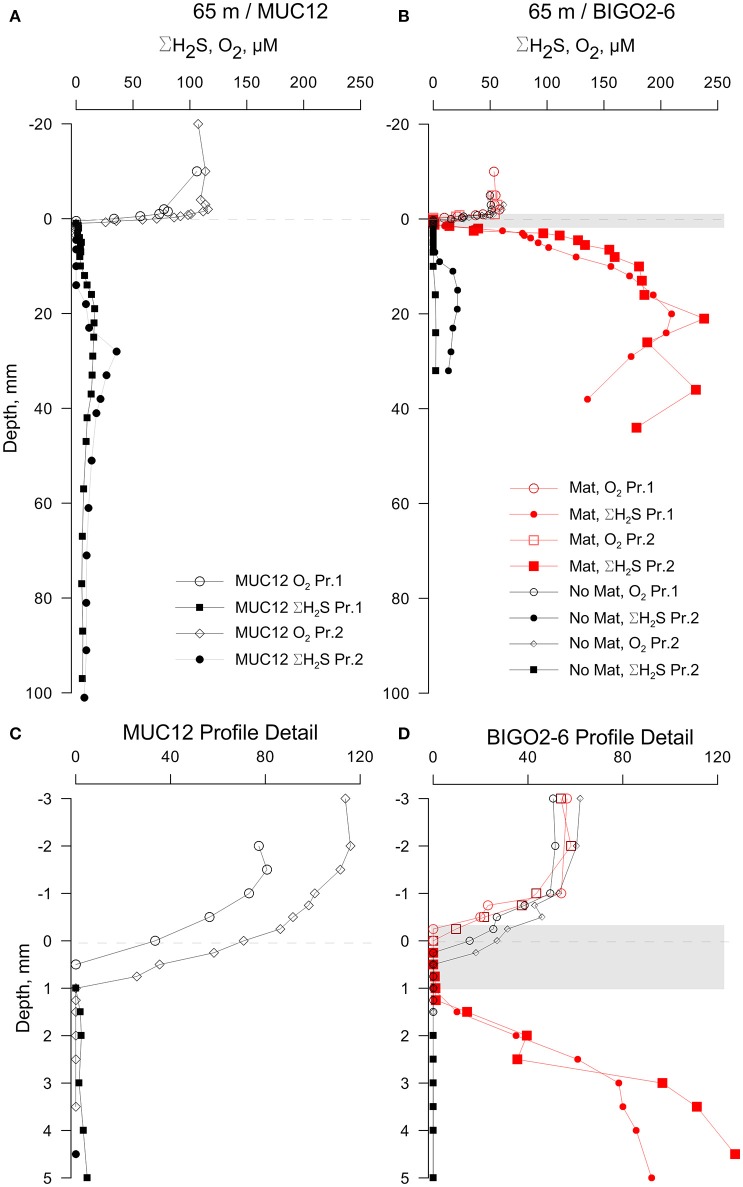
**Results of the voltammetric profiling in sediments from 65 m depth. (A)** Profiles show only a moderate enrichment of sulfide in sediments with no visible bacterial mats while the BIGO cores from the same depth **(B)** had partial coverage of bacterial mats (shown as a gray band—the thickness is approximate) and a high sulfide flux. **(C,D)** shows enlarged views of the sediment-water interface shown in **(A,B)**, respectively. “Pr1” and “Pr2” refer to replicate profiles. Negative depths denote distance below the sediment-water interface (dashed line).

While the porewater profiles on sectioned cores revealed important trends through the basin, higher resolution microelectrode profiles resolved the fine scale processes that determine the fluxes at the sediment-water interface. Microprofiles from the permanently oxic station (65 m) revealed a quite sharp O_2_ gradient across the sediment-water interface. Indeed, this was the only sampling location that had a detectable oxygen gradient across the sediment-water interface (Figure 3). Oxygen penetrated only to about 1 mm here, below which ΣH_2_S gradually increased to a maximum concentration of 36 μM at about 30 mm depth. The sub-core from the BIGO chamber obtained from the 65 m–station had a patchy distribution of microbial mats. The core subsampled from a non-microbial mat area from one the BIGO chambers had similar porewater chemistry as the MUC. However, the sediment subcore sampled over a white microbial mat had significantly higher levels of ΣH_2_S with depth, with a subsurface maximum concentration of 238 μM at 21 mm. The presence of the mat also modified the O_2_ gradients such that O_2_ was depleted right at the sediment-water interface just above the mat (due to high sulfide flux) but in the absence of a mat O_2_ penetrated to about 0.5 mm depth (no sulfide flux) (red and black curves in Figures 3C–D). In the profile with the mat, the actual oxygen depletion starts about 0.5 mm above the mat, which may point to additional oxidation pathways above the mat. The oxygen gradient may also have been modified by non-steady state effects due to increasing thickness of the DBL (see methods). Our assignment of the mat location should also be viewed with a ± 0.5 mm error. Still, we hold the view that the non-overlap of the O_2_ and ΣH_2_S gradients reflect natural conditions because O_2_ diffuses into the top of the mat whereas H_2_S diffuses into the bottom. As will be elaborated later, within the mat H_2_S can be oxidized to intermediate S oxidation states, with the additional possibility of abiotic Fe-H_2_S cycling. This could also contribute to the spatial separation of O_2_ and H_2_S gradients, as has been demonstrated in bioturbated sediments previously (e.g., Jørgensen and Nelson, 2004). Either way, our *in situ* flux measurements strongly indicate that H_2_S oxidation is occurring, and that H_2_S is not reaching the bottom water (see below).

**Figure 4:**
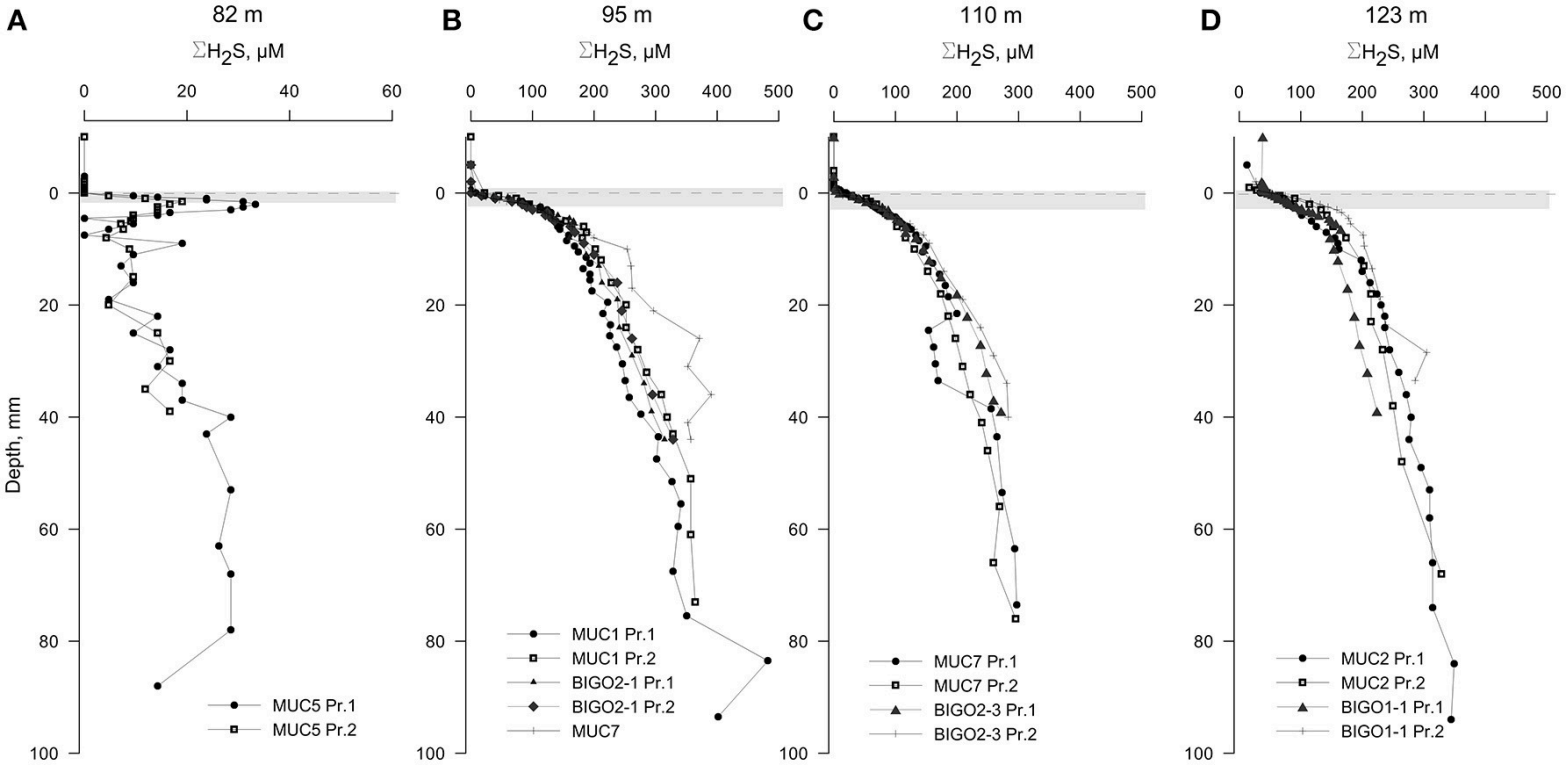
**Voltammetric profiles in sediments located along the HTZ in the EGB**. Except for the 82 m site **(A)**, all cores **(B–D)** displayed high sulfide fluxes near the sediment-water interface. “Pr1” or “Pr2” refers to replicate profiles. Positive depths denote distance below sediment-water interface (dashed line). The approximate locations of the bacterial mats are indicated by a gray bands. Note the expanded concentration scale in **(A)**.

**Figure 5:**
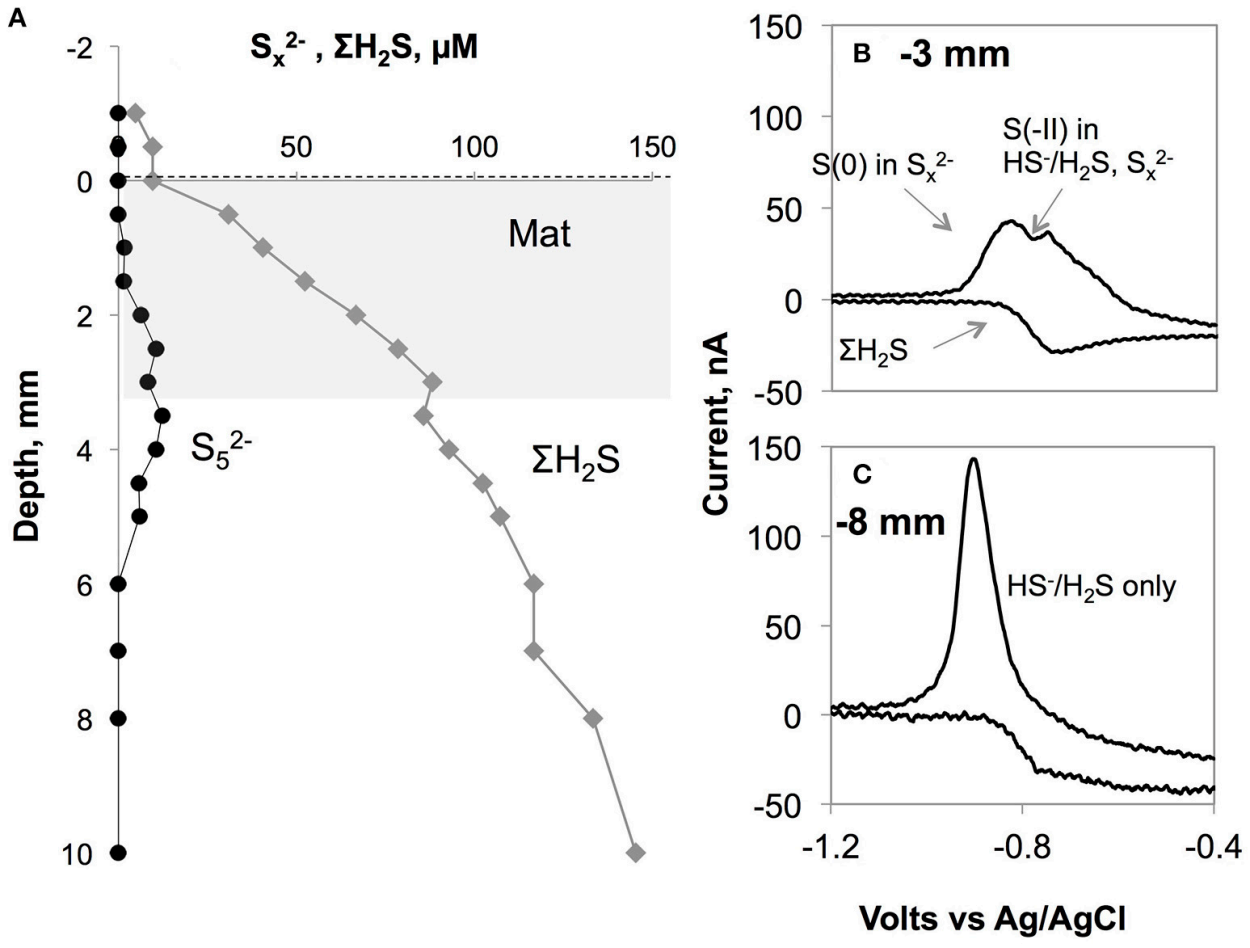
**(A)** Polysulfide concentration (assumed to be ${\text{S}}_{5}^{2-}$) plotted along with total sulfide levels in the core from BIGO2-3 from 110 m depth. The gray area shows the approximate location of the white bacterial mats. **(B)** shows a typical voltammogram with the co-existence of free sulfide and polysulfides. The double peak arises from the two different oxidation states existing in a ${\text{S}}_{\text{x}}^{2-}$ molecule (all S have 0 valence except for the terminal S with −2 valence). The more negative peak in the forward scan corresponds to (x-1) zerovalent sulfur atoms in the polysulfides while the other peak is the sum of free sulfide plus the terminal S(-II) in ${\text{S}}_{\text{x}}^{2-}$. On the backward scan, a wave-like signal can be used for calculation of total dissolved sulfide. This signal was obtained within or just underneath the *Beggiaota* mats. Similar signals were found within the 0–5 mm depth interval in sediments from 95, 110, and 123 m depths. The scan rate was 1000 mV s^−1^ As shown in **(C)**, below ca. 5 mm the double peak disappeared giving rise to the single peak typical for H_2_S/HS^−^.

The oxygen levels in the overlying waters of the cores from 82, 95, 110, and 123 m were undetectable by the voltammetric system (<20 μM). Still, micromolar levels of O_2_ are known to be intermittently available in this depth range (Meyer et al., 2014; Noffke et al., 2016). Dissolved sulfide was the only voltammetric species detected whereas all other analytes remained below detection limits therefore not plotted (Figure 4). The core from 82 m had relatively low levels of ΣH_2_S with two subsurface maxima of about 30 μM at 2 and 70 mm. The two maxima could be due to elevated sulfate reduction rates in the very surface and deeper parts. As in all other HTZ cores, a sharp ΣH_2_S gradient was present on the top several millimeters (Figure S2). ΣH_2_S profiles in the 95, 110, and 123 m cores also indicate high fluxes of ΣH_2_S (highest concentration of about 400 μM at 100 mm depth), which is completely consumed in the top mm of the sediment, likely by the mediation of the sulfide-oxidizing microbial community. Strong evidence in support of this idea is the consistent appearance of a “double peak” voltammetric polysulfide signal (Figure 5)—which probably originates from the bacterially generated zerovalent sulfur (see Microbially Mediated Sulfide Oxidation as a Major Driver of the Benthic Filter).

**Figure 6:**
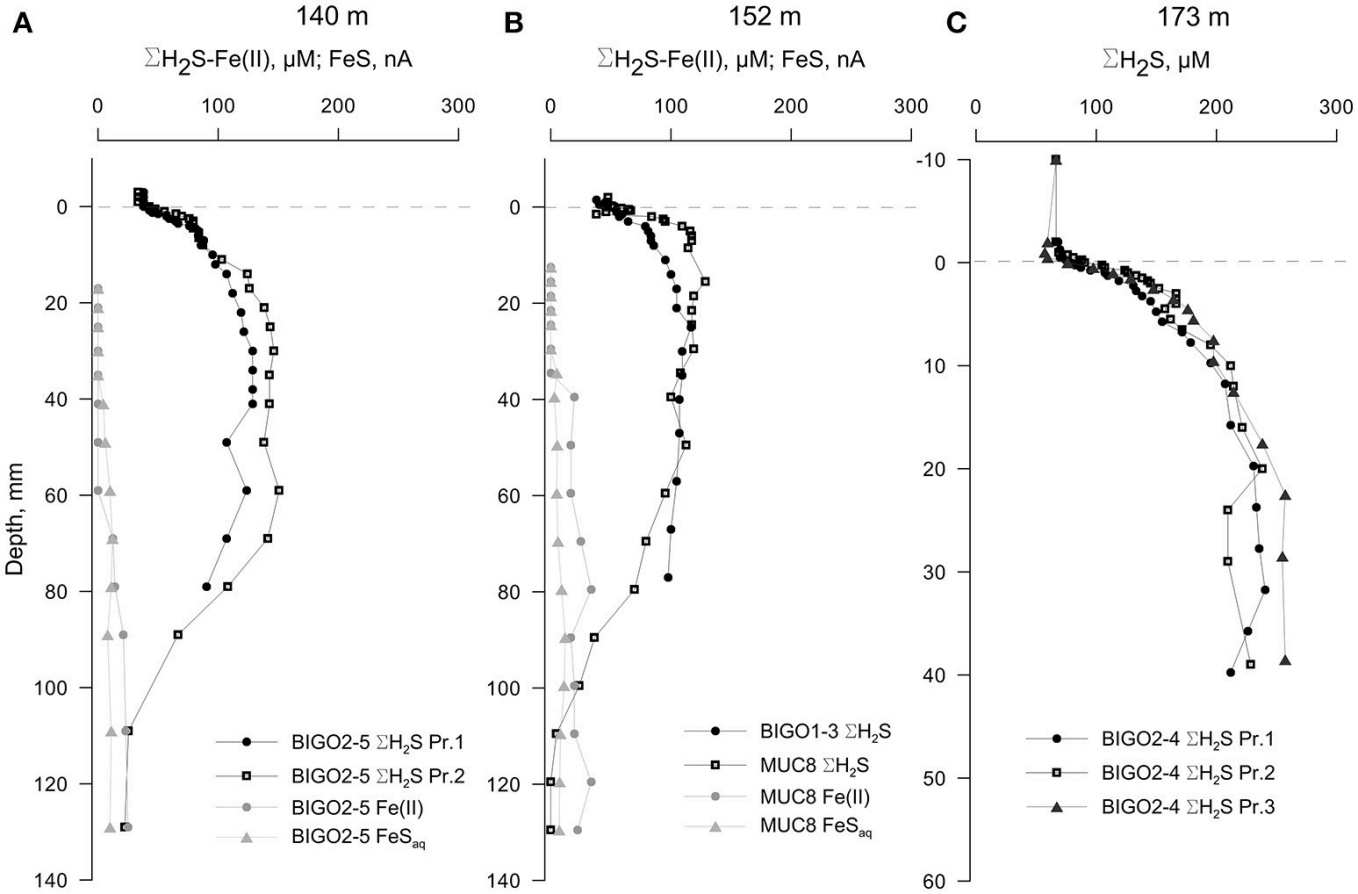
**Voltammetric profiles in sediments underlying anoxic-sulfidic waters in the EGB**. **(A)** 140 m core, **(B)** 152 m core and **(C)** 173 m core. “Pr1” and “Pr2” refer to replicate profiles. Positive depths denote distance below the sediment-water interface (dashed line). Note that the depth-axis in **(C)** has an enlarged scale. The redox-active parameters that were below detection limits are not plotted.

The cores from water depths larger than 123 m had detectable levels of ΣH_2_S in the overlying waters (Figure 6, also Figure S3). ΣH_2_S increased to a subsurface maximum of around 120 μM in both the 140 and 152 m cores, and the levels of ΣH_2_S decreased to zero after typically 100 mm depth which denotes the top of the clay layer. The decrease in ΣH_2_S was accompanied by the appearance of dissolved iron as well as voltammetric signals for aqueous FeS (see Figure S4 for an example voltammogram). Presumably, then, ΣH_2_S is precipitated as iron-sulfide particulates at this interface (e.g., Holmkvist et al., 2014). The profiles from the 174 m core were penetrated only until 40 mm depth; thereafter the electrochemical signals repeatedly went offscale due to very high sulfide concentrations (Figure 2D).

### Benthic sulfide fluxes across the transect

**Figure 7:**
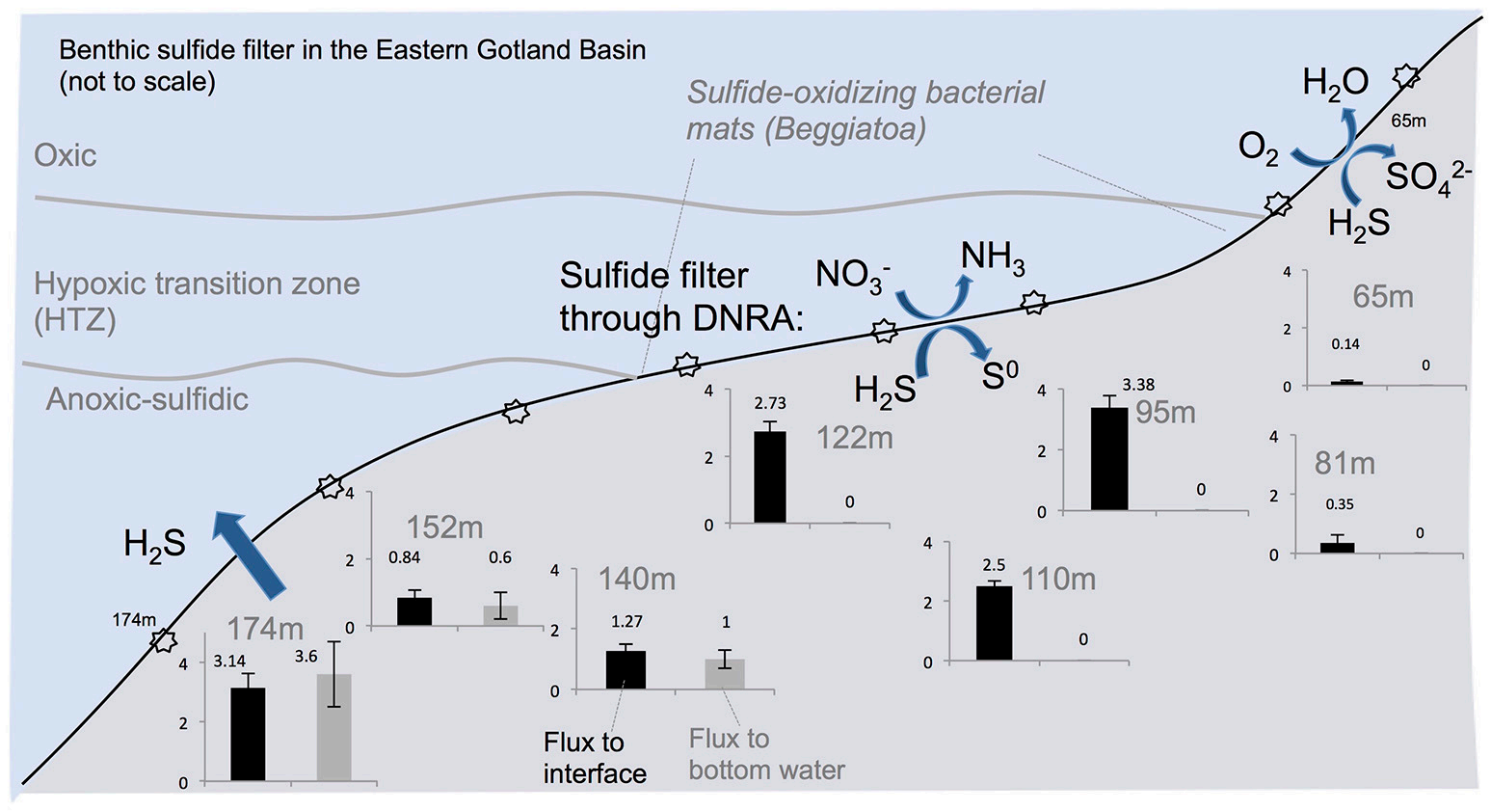
**Summary of the benthic sulfide filter in the Eastern Gotland Basin, Baltic Sea. Starbursts indicate sampling locations which are given in Table 1**. Black bars denote ΣH_2_S flux to the sediment-water interface and the gray bars indicate flux (mmol m^−2^ day^−1^) to the bottom water. The gray bars are zero where the benthic sulfide filter is active.

The calculated diffusive fluxes at the sediment-mat interface indicate that ΣH_2_S consumption was between 2.5 and 3.4 mmol m^−2^ day^−1^ for the sediments located in the HTZ (Table 2 and Figure 7). This flux enters the base of conspicuous white bacterial mats, thus alluding to a microbial mediation of sulfide oxidation. No ΣH_2_S efflux to the overlying waters was detected during microprofiling. At deeper locations without microbial mats, sulfide instead enters the water column, with the highest efflux occurring at the 173 m site (3.14 mmol m^−2^ day^−1^). Due to lower sampling resolution compared to microprofiling, the diffusive fluxes calculated from the data (upper 10 cm) on sectioned cores were much lower. For instance, fluxes at the HTZ stations were 0.8–1.3 mmol m^−2^ day^−1^ and 1.9 mmol m^−2^ day^−1^ at the 173 m station.

**Table 2:** **Summary of microelectrode-derived fluxes in surface sediments**.

**Water depth**	**Component**	**Flux, mmol m^−2^ day^−1^**	**Depth Interval, mm**
65m no mat	O_2_	−4.83 ± 1.16	+1 to −0.5
65m no mat	ΣH_2_S	+0.14 ± 0.05	−5 to −20
65m with mat	O_2_	−7.59 ± 1.89	+1 to 0
65m with mat	ΣH_2_S	+3.41 ± 0.55	−1 to −5
82m	ΣH_2_S	+0.35 ± 0.27	0 to −3
95m	ΣH_2_S	+3.38 ± 0.40	0 to −5
110m	ΣH_2_S	+2.50 ± 0.17	0 to −5
123m	ΣH_2_S	+2.73 ± 0.30	0 to −5
140m	ΣH_2_S	+1.27 ± 0.22	0 to −4
140m	ΣH_2_S	−0.19 ± 0.04	−60 to −110
140m	Fe(II)	+0.008 ± 0.001	−60 to −130
152m	ΣH_2_S	+0.84 ± 0.23	0 to −5
152m	ΣH_2_S	−0.14 ± 0.02	−40 to −110
152m	Fe(II)	+0.01 ± 0.003	−40 to −120
173m	ΣH_2_S	+3.14 ± 0.48	0 to −5

*Positive fluxes are directed out of the sediment*.

*See the text for details of flux and error calculations. No flux was measurable for the components that are not listed at a given depth*.

High sulfide fluxes not only occurred in the HTZ and anoxic basin sediments. Despite relatively low levels of ΣH_2_S in sediments from 65 m depth, the core sampled from the chamber of the BIGO lander deployed at the same location was partially covered with bacterial mats. As shown in Figure 3, these mats are characterized by a high ΣH_2_S gradient such that the ΣH_2_S flux toward the sediment-water interface within a mat increased to 3.4 ± 0.3 mmol m^−2^ day^−1^ whereas in an adjacent non-mat area the ΣH_2_S flux was much weaker (0.14 ± 0.02 mmol m^−2^ day^−1^). This localized sulfide source apparently increases the oxygen consumption in the sediment such that in the non-mat area the O_2_ flux into the sediment was 4.83 ± 1.16 mmol m^−2^ day^−1^ compared to 7.59 ± 1.89 mmol m^−2^ day^−1^ in the mat. Hence, the increase in the sulfide flux was matched by an almost equal rate of O_2_ consumption.

The sulfide fluxes obtained from the benthic lander deployments also revealed a similar overall pattern (Table 3). No sulfide flux was measurable from the HTZ sediments to bottom waters due to the benthic microbial sulfide filter. Deeper cores had sulfide fluxes to the bottom waters in the range of 5.3–10.2 mmol m^−2^ day^−1^; at least a factor of three higher than those obtained by microprofiling. This may be caused by either seafloor heterogeneity or because the large footprint of the benthic chamber provides a more complete picture of benthic fluxes than single point microprofiling measurements. Comparing our findings with previously published sulfide flux measurements to the bottom water of the EGB, we observed similar values to Noffke et al. (2016) who reported a maximum flux of 3.5 mmol m^−2^ day^−1^ using lander-based incubations in 2013 and to McGinnis et al. (2011) who reported an average flux of 1.9 mmol m^−2^ day^−1^ using an eddy correlation instrument deployment at 192 m depth.

**Table 3:** **Benthic fluxes in the EGB measured using benthic landers (mmol m^**−2**^ day^**−1**^)**.

**Deployment**		**Water depth (m)**	**Incubation time (h)**	**ΣH_2_S (mmol m^−2^ day^−1^)**
BIGO-II-6	CH1	65	30.0	bdl
				bdl
		CH2		
BIGO-I-2	CH1	80	31.5	bdl
	CH2			bdl
BIGO-II-1	CH1	96	30.0	bdl
	CH2			bdl
BIGO-II-3	CH1	110	31.0	bdl
	CH2			bdl
BIGO-I-6	CH1	110	29.0	bdl
				bdl
		CH2		
BIGO-I-5	CH1	123	30.0	bdl
				bdl
		CH2		
BIGO-I-4	CH1	123	30.0	nd
	CH2			bdl
BIGO-I-1	CH1	124	30.0	bdl
				bdl
		CH2		
BIGO-II-5	CH1	140	29.0	7.61
				5.27
		CH2		
BIGO-I-3	CH1	152	35.0	3.24
	CH2			4.11
BIGO-II-4	CH1	173	31.0	10.15
	CH2			9.81

*Fluxes for both chambers (CH1, CH2) of each lander deployment are provided*.

*Positive fluxes are directed out of the sediment. (bdl, below detection limit; nd, not determined)*.

## Discussion

### Microbially mediated sulfide oxidation as a major driver of the benthic filter

The elevated sulfide fluxes for the top 5 mm of the sediments determined from high-resolution profiling along with the presence of microbial mats together indicate a role for biological sulfide consumption and mitigation of sulfide efflux to the bottom waters, i.e., a microbial benthic filter. As suggested by Noffke et al. (2016), these mats are dominated by sulfide-oxidizing *Beggiatoa* sp. (also Sommer, personal observations during 2013 August and H. Schulz, pers. comm.). The HTZ waters of the EGB have variable but low levels of oxygen and nitrate (Meyer et al., 2014), both of which can be used as an electron acceptor for sulfide oxidation:

(3) $$\begin{array}{l} 2{\text{HS}}^{-}+\text{½}{\text{O}}_{2}\to 2S^{0}+{\text{H}}_{2}\text{O} \end{array}$$

(4) $$\begin{array}{l} 4{\text{HS}}^{-}+{\text{NO}}_{3}^{-}+6{\text{H}}^{+}\to {\text{NH}}_{4}^{+}+4S^{0}+3{\text{H}}_{2}O \end{array}$$

The latter equation, when coupled to chemosynthetic CO_2_ fixation, is described as dissimilatory reduction of nitrate to ammonium (DNRA; Jørgensen and Nelson, 2004). Similar to denitrification, DNRA is a pathway that eliminates ${\text{NO}}_{3}^{-}$, but unlike denitrification, fixed N is not lost as N_2_ but is retained as ${\text{NH}}_{4}^{+}$. The study by Noffke et al. (2016) indicated that DNRA was pronounced in HTZ sediments sampled in 2013, yielding elevated release of ${\text{NH}}_{4}^{+}$ to bottom waters (0.68–1.10 mmol m^−2^ day^−1^) with equal ${\text{NO}}_{3}^{-}$ fluxes in the opposite direction (see Noffke et al., 2016, Figure 5). The microelectrode-derived sulfide fluxes consumed by the mat in the HTZ area, ranging from 2.78 to 3.38 mmol m^−2^ day^−1^, is nearly 3–4 times the ${\text{NH}}_{4}^{+}$ fluxes, hence supporting the view that the driver of sulfide oxidation in the HTZ is DNRA. *Beggiatoa* and other sulfide oxidizers can use sulfide also as the electron donor for CO_2_ fixation. Thus, the ratio would allow for the complete oxidation of a fraction of the sulfide pool to sulfate.

The end product of equations 3 and 4, elemental sulfur (S^0^), has been frequently observed within the vacuoles of the *Beggiatoa* bacteria (e.g., Pasteris et al., 2001; Schwedt et al., 2012). The dissolved precursor to particulate elemental sulfur (S_8_) is a polysulfide molecule (${\text{S}}_{\text{x}}^{2-}$). When the chain length exceeds 8 the molecule forms an S_8_ ring, releasing HS^−^ to solution. Polysulfides are a well-known intermediate in the oxidation of ΣH_2_S (e.g., Lichtschlag et al., 2013) and have been documented in the pelagic chemocline of the Gotland Basin (Kamyshny et al., 2013). These species are also electroactive and in our dataset voltammetric signals for them (one example shown in Figure 5) were detected in the vicinity of the mats between 1 and 5 mm depth within the surface sediment. Given that polysulfides are transient chemical products, it seems likely that the mats were actively oxidizing sulfide at the time of microprofiling.

The quantification of voltammetric polysulfide signals is rather difficult due to the undetected chemical speciation of these species due to lack of appropriate standards. Despite this drawback, an estimate is given in Figure 5 for a mat located over the sediments at 110 m depth following the method of Rozan et al. (2000). This method takes advantage of the double peak in the sulfide forward wave in the voltammogram. The double peak is due to the two oxidation states existing in a ${\text{S}}_{\text{x}}^{2-}$ molecule (0 as in elemental sulfur but with a terminal S with -2 valence), such that it is possible to discriminate these two states with the fast voltammetric scan rates that were employed in this study (>1000 mV s^−1^) (Luther et al., 2001). The more negative peak in Figure 5B corresponds to (x-1) zerovalent sulfur atoms in the polysulfides while the other peak is the sum of free sulfide plus the terminal S(-II) in ${\text{S}}_{\text{x}}^{2-}$. If the polysulfides are assumed contain 5 sulfur atoms on average (likely for a sedimentary pH range of 7–8, Kamyshny et al., 2004, 2008) and assuming that their calibration slope is the same as that of free sulfides, then we can infer a maximum of 12 μM polysulfides, accounting for 14% of total sulfide.

Similar voltammetric polysulfide signals were obtained in intertidal mats (Glazer et al., 2002), diffuse flow habitats in deep-sea hydrothermal vents (Gartman et al., 2011) and in laboratory studies of microbial S oxidizers (Sun et al., 2009). More recently, using single-cell Raman spectroscopy, Berg et al. (2014) detected intracellular polysulfide storage (in addition to S_8_) in *Beggiatoa* cultures. Aside from their formation during sulfide oxidation, polysulfides can also be derived from the cleavage of the S-S bonds in the S_8_ rings through intracellular enzymes (Berg et al., 2014) or chemically by the attack of HS^−^ (Kamyshny and Ferdelman, 2010). Taking these studies as a vantage point, the location of the subsurface polysulfide maximum (Figure 5A) in our results can be affected by two interrelated mechanisms. First, the polysulfides could be produced within the bacterial cells as a result of the utilization of the intracellular elemental sulfur and subsequent release of produced polysulfides to the porewater. Second, as the oxidation product of Equations 3, 4, elemental sulfur can be excreted from bacteria after which polysulfides can abiotically form in the porewater. For the ${\text{S}}_{5}^{2-}$ species discussed above, this reaction can be written as (Kamyshny et al., 2008):

(5) $$\begin{array}{l} {\text{HS}}^{-}+4{\text{ S}}^{0}\leftarrow \to {{\text{S}}_{5}}^{2-}+{\text{H}}^{+} \end{array}$$

Shipboard microscopy observations of the bacteria (Sommer, unpubl.) revealed that their vacuoles were full of sulfur particles, implying that the release of newly produced sulfur to the porewaters is likely. The more reducing conditions below the mat may help stabilize and accumulate polysulfides, resulting in the subsurface maximum shown in Figure 5. Such a release of sulfide originating from the sulfur inclusions within a culture of *Beggiatoa* was demonstrated by Schwedt et al. (2012). Here, the sulfide release was due to the reduction of zerovalent sulfur by polyhydroxyalkanoates (PHA) inclusions that were also synthesized in the cell during CO_2_ fixation. Schwedt et al. (2012) noted “H_2_S” release as a product, but it could have been the case that ${\text{S}}_{\text{x}}^{2-}$, being more reduced than S^0^, could also form during this process. However, we cannot rule out the participation of a microbial consortium rather than Beggiatoa alone, that is, zero-valent sulfur may be the product of other sulfur oxidizing bacteria (e.g., Thiobacillus) that co-inhabit the mat. Future work will shed more light on this issue, and our findings are in parallel with emerging evidence supporting the idea that these sulfur intermediates can play important role in the benthic sulfur turnover in low-O_2_ environments.

High sulfide oxidation rates have also been detected in other reducing habitats where microbial mats contribute to the formation of steep sulfide gradients. The fluxes, however, can vary within one order of magnitude. The pioneer microsensor study of Revsbech and Jørgensen (1983) found that in a shallow water setting *Beggiatoa* mat, the sulfide flux was 38 mmol m^−2^ day^−1^ whereas Preisler et al. (2007) reported a value of 5 mmol m^−2^ day^−1^ in a different coastal sediment. Recent *in-situ* microsensor studies from cold seeps at mud volcanoes yielded even higher rates. For example, in the *Beggiatoa* habitat in the Haakon Mosby mud volcano (Barents Sea), de Beer et al. (2006) measured a flux of 18.7 mmol m^−2^ day^−1^ while Grünke et al. (2011) gave a flux of 40 mmol m^−2^ day^−1^ in a similar habitat in the Amon Mud Volcano in the Nile Fan (Eastern Mediterranean). An important contributor to these high fluxes is the sulfide production fueled by the anaerobic oxidation of methane coupled to sulfate reduction near the sediment-water interface. In continental margin sediments under upwelling areas, sulfide fluxes (associated with bacterial mats) mostly remain below 15 mmol m^−2^ day^−1^ (Zopfi et al., 2008; Sommer et al., 2016). These systems, as opposed to cold seeps, are dominated by the diffusive fluxes of sulfide generated through organic matter degradation. Taken together, our fluxes from the EGB remain at the lower end of the reported fluxes, closer to the estimates for continental margins under upwelling areas. One common finding of the above-mentioned studies is that microbial residents of sharp sulfide gradients such as *Beggiatoa* use ${\text{NO}}_{3}^{-}$ as well as O_2_ as an oxidant for energy acquisition for the production of biomass. The ability of intracellular nitrate storage gives these bacteria a significant ecological advantage in redox transition waters such as those in the EGB where O_2_ and ${\text{NO}}_{3}^{-}$ are intermittently available. Sulfide, on the other hand, is generally not limiting in these organic-rich habitats. These dynamic biogeochemical conditions can result in the widespread presence of sulfide-oxidizing benthic bacterial mats which increases the sulfide retention capacity of the seafloor (Schulz and Jørgensen, 2001). A newly discovered group of “cable bacteria,” without forming visible mats, have also been shown to oxidize sulfide in the upper sediment by shuttling electrons via cm-long filaments that connect otherwise vertically separated oxygen and sulfide gradients (Pfeffer et al., 2012; Seitaj et al., 2015). While they remain to be discovered in the EGB, we currently neglect their contribution to the benthic sulfide filter in the EGB because our measured sulfide gradients are very sharp at the sediment-water interface and lack a vertical separation from the oxygen gradient.

### Regional implications of benthic sulfide oxidation in the Baltic Sea

The findings reported here bring forward the question of whether a similar benthic sulfide filter operates in other areas of the Baltic Sea with hypoxic-anoxic bottom waters. Towed camera observations during our cruise (ALK 422) revealed that the seafloor under the HTZ in the other parts of the Gotland Basin was also covered by extensive bacterial mats (Pfannkuche and Sommer unpubl.). Hence, sulfide oxidation at the seafloor below the redox transition zone may be important on a regional scale. Hannson et al. (2013) and Noffke et al. (2016) estimated the area of Baltic Proper seafloor under permanently anoxic waters (>120 m depth) as 18954 km^2^ and under HTZ waters (80–120 m) as 47320 km^2^. Using these areal estimates and the average of the measured fluxes (2.87 mmol m^−2^ day^−1^) for the 80–120 m depths and the 173 m flux (3.14 mmol m^−2^ day^−1^), we estimate that a total of 2281 kton S yr^−1^ of this sulfide flux occurs at the sediment surface (0–5 mm) below a depth of 80 m. Of this, about 695 kton S yr^−1^ enters the water column below 120 m. The eventual fate of the flux is oxidation in the water column chemocline (Kamyshny et al., 2013). The remaining 1586 kton S yr^−1^, corresponding to 70% of total flux occurring in the sediments beneath 80 m water depth, is removed in the benthic interface located under the HTZ. This analysis bears obvious uncertainties, but it suffices to highlight the important ecosystem service provided by these benthic microbial communities in the Baltic Sea.

### FeS formation in the deep water sediments

In addition to sulfide oxidation at the sediment-water interface, some cores exhibited a deeper sulfur sink. The 140 and 152 m cores had a subsurface sulfide peak after which voltammetric signals for Fe(II) and FeS appeared and increased in intensity with depth. These opposing geochemical gradients also corresponded to the abrupt shift from a laminated, organic-rich surface sediment layer toward a more homogeneous, gray, low porosity layer. This points to a deep source of Fe(II) arising from the reduction of particulate Fe(III), which was reported to be in high concentration in earlier depositional periods (i.e., Ancylus Lake, Boettcher and Lepland, 2000; Holmkvist et al., 2014). We observed that FeS formation is clearly occurring in the lower part of the organic-rich sulfidic layer (Figure 6). Therefore, in addition to the removal of sulfide mediated by sulfide oxidizing bacteria on the surface sediments of the chemocline sediments, FeS precipitation acts as a second sink for sulfide (Rickard and Luther, 2007):

(6) $$\begin{array}{l} {\text{Fe}}^{2+}+{\text{HS}}^{-}\to {\text{FeS}}_{\text{aq}}+{\text{H}}^{+} \end{array}$$

When compared with the downward ΣH_2_S fluxes at 140 and 152 m cores (−0.19 and −0.14 mmol m^−2^ day^−1^), the upward Fe(II) flux of 0.01 mmol m^−2^ day^−1^, is too low to account for sulfide consumption. This indicates that H_2_S must be actively consumed in further reactions with aqueous or particulate FeS to precipitate pyrite, FeS_2_ (Yücel et al., 2010);

(7) $$\begin{array}{l} {\text{FeS}}_{\left(\text{s}\right),\left(\text{aq}\right)}+\text{HS}{-}_{\left(\text{aq}\right)}\to {\text{FeS}}_{2\left(\text{s}\right)}+\text{½}{\text{H}}_{2\left(\text{g}\right)} \end{array}$$

Solid phase pyrite or FeS measurements were not conducted in this study but other works such as that by Boettcher and Lepland (2000) and Holmkvist et al. (2014) already identified an iron sulfidation interface at the Anyclus-Littorina transition where the laminated organic-rich Littorina sediments are enrich in pyrite whereas the top part of the lacustrine layers are enrich in FeS. A similar iron-sulfidation front may exist in our 140 and152 m cores at a depth of 10–15 cm only, where the transition to a gray, homogeneous layer is visible.

A likely scenario for the appearance of the gray homogeneous layer close to the sediment-water interface is that these sediments might have deposited during the freshwater Ancylus Lake (Andren et al., 2000). A first support of this idea is the very low POC-PS levels (Figure 2), which has been reported as a characteristic of pre-Littorina deposition (Boettcher and Lepland, 2000). However, this finding is rather unexpected since the presumed Ancylus Lake sediments were located 200–400 cm below the sediment-water interface (Boettcher and Lepland, 2000) whereas our results from 140 to 152 m cores suggest that this can also happen at very shallow sediment depths. (i.e., 10–20 cm). This can be explained by the fact that earlier studies focusing on Ancylus-Littorina transition sampled from the deepest parts of the Baltic, where the accumulation rates are known to be highest (Vallius and Kunzendorf, 2001; Hille et al., 2006). In the EGB margin, especially between 100 and 150 m depth, near bottom currents are reported to be highest (Emeis et al., 1998; Hagen and Feistel, 2004), limiting the accumulation of recent material. Seismic studies in the EGB also hint at a very variable lateral distribution of depositional layers (Emeis et al., 1998), supported by reports on variable ^210^Pb-based mass accumulation (Hille et al., 2006) and organic carbon burial rates (Winogradow and Pempkowiak, 2014). These considerations point to the possibility that the Ancylus Lake sediments may already be present at quite shallow depths at certain locations along the basin margin. In addition to the possibility that the gray homogeneous layer had a lake origin, it may also be the case that they formed during an oxic era in the Littorina Sea when the bottom sediments were bioturbated, presumably during the latest presumably “oxic” event, which occurred between ca 1200 and 1900 AD (Conley et al., 2009). Still, the very low POC levels in these depositional layers argue against a marine origin. Regardless of their provenance, these erosion bottom sediments (Jonsson et al., 1990) that underly the most recent organic-rich deposits can be a near-seafloor dissolved iron source and act as a sulfide sink in the deep basins of the Baltic.

### Baltic Sea benthic sulfur cycle provides an important feedback to the pelagic ecosystem

Our combined approach using porewater measurements on sectioned cores, voltammetric microelectrode profiling and *in situ* lander incubations confirmed the presence of a highly efficient microbial hydrogen sulfide filter in the EGB. We have also found that sulfide accumulation was limited to a recent sedimentary layer that was rich in organic carbon. Not only the deep-water sediments but also the shallow water sediments contained dissolved sulfide as evidenced by patches of bacterial mats at these shallow depths. Sediments of both the deep basin and HTZ zones were highly sulfidic—however the sulfide flux was effectively consumed in the vicinity of the extensive bacterial mats beneath the HTZ. The dissolved iron flux reaching the near-surface sediments at 140–152 m cores apparently plays a role in limiting the sulfide efflux toward the water column, although quantitatively they are not be as important as the microbial filtering.

The benthic sulfur cycle in the Baltic Sea is tightly coupled to the cycles of nitrogen especially via DNRA (Noffke et al., 2016). Here, sulfide oxidation with nitrate as an electron acceptor yields elevated ammonium fluxes to bottom water (via DNRA, Noffke et al., 2016; Sommer et al., 2017) thereby leading to the retention of fixed nitrogen in the system. Understanding the intertwined cycling of C, N, and S with an integrated approach will be more important since the Baltic Sea, similar to other hypoxic coastal environments, is undergoing an accelerated change (Carstensen et al., 2014) where the intensification of the hypoxia may also elevate sulfide accumulation. In the light of these projections, whether or not the benthic sulfide filter will remain as efficient as it is at present remains an open question.

## Author contributions

MY, SS, AD, OP designed the study; OP and SS coordinated ship operations, lander deployments and sampling; MY, SS, AD took and processed samples; MY performed microelectrode measurements and analyses, MY, SS, AD performed data analyses and calculations; MY, SS, AD, and OP wrote the manuscript.

## Funding

This research was funded by the Helmholtz Alliance Robotic Exploration of Extreme Environments (ROBEX) and partly funded by the Sonderforschungsbereich 754 “Climate—Biogeochemistry Interactions in the Tropical Ocean” (http://www.sfb754.de) which is supported by the Deutsche Forschungsgemeinschaft. M. Yücel also acknowledges start-up funds from Turkish Scientific and Technological Research Council (TUBITAK 2232 Program, Project No: 115C090) and from Project DEKOSIM - National Excellence Centre for Marine Ecosystem and Climate Research, funded by the Ministry of Development of Turkey, which provided support during manuscript drafting and revision stages.

### Conflict of interest statement

The authors declare that the research was conducted in the absence of any commercial or financial relationships that could be construed as a potential conflict of interest.

## Supplementary material

Figure S1 **Photographs of the MUC cores taken from the transect**.

Figure S2 **Zoomed (top 10 mm) version of the graphs in Figure 4**.

Figure S3 **Zoomed (top 10 mm) version of the graphs in Figure 6**.

Figure S4 **Representative voltammetric scan showing the coexistence of Fe(II) and FeS (from the 152 m-core, 100 mm)**.

## References

[B1] Andren E. Andren T. Sohlenius G. (2000). The Holocene history of the southwestern Baltic Sea as reflected in a sediment core from the Bornholm Basin. Boreas 29, 233–250. 10.1111/j.1502-3885.2000.tb00981.x

[B2] Berg J. S. Schwedt A. Kreutzmann A.-C. Kuypers M. M. M. Milucka J. (2014). Polysulfides as intermediates in the oxidation of sulfide to sulfate by *Beggiatoa* spp. Appl. Environ. Microbiol. 80, 629–636. 10.1128/AEM.02852-13 24212585 PMC3911116

[B3] Boettcher M. E. Lepland A. (2000). Biogeochemistry of sulfur in a sediment core from the west-central Baltic Sea: evidence from stable isotopes and pyrite textures. J. Mar. Syst. 25, 299–312. 10.1016/S0924-7963(00)00023-3

[B4] Boudreau B. P. (1997). Diagenetic Models and Their Implementation, Modelling Transport and Reactions in Aquatic Sediments. Berlin: Springer-Verlag.

[B5] Brendel P. Luther G. W. (1995). Development of a gold-amalgam voltammetric microelectrode for the determination of dissolved Fe, Mn, O2 and S(-2) in porewaters of marine and freshwater sediments. Environ. Sci. Technol. 29, 751–761. 10.1021/es00003a024 22200285

[B6] Bura-Nakic E. Krznaric D. Jurasin D. Helz G. R. Ciglenecki I. (2007). Voltammetric characterization of metal sulfide particles and nanoparticles in model solutions and natural waters. Anal. Chim. Acta 594, 44–51. 10.1016/j.aca.2007.04.065 17560384

[B7] Carstensen J. Andersen J. H. Gustafsson B. G. Conley D. J. (2014). Deoxygenation of the Baltic Sea during the last century. Proc. Natl. Acad. Sci. U.S.A. 111, 5628–5633. 10.1073/pnas.1323156111 24706804 PMC3992700

[B8] Conley D. J. Björck S. Bonsdorff E. Carstensen J. Destouni G. Gustafsson B. G.. (2009). Hypoxia-related processes in the Baltic Sea. Environ. Sci. Technol. 43, 3412–3420. 10.1021/es802762a 19544833

[B9] Dale A. W. Bertics V. J. Treude T. Sommer S. Wallmann K. (2013). Modeling benthic-pelagic nutrient exchange processes and porewater distributions in a seasonally-hypoxic sediment: evidence for massive phosphate release by Beggiatoa? Biogeosciences 10, 629–651. 10.5194/bg-10-629-2013

[B10] Dale A. W. Sommer S. Ryabenko E. Noffke A. Bohlen L. Wallmann K. (2014). Benthic nitrogen fluxes and fractionation of nitrate in the Mauritanian oxygen minimum zone (Eastern Tropical North Atlantic). Geochim. Cosmochim. Acta 134, 234–256. 10.1016/j.gca.2014.02.026

[B11] de Beer D. Sauter E. Niemann H. Kaul N. Witte U. Schlu M. (2006). *In-situ* fluxes and zonation of microbial activity in surface sediments of the Hakon Mosby Mud Volcano. Limnol. Oceanogr. 51, 1315–1331. 10.4319/lo.2006.51.3.1315

[B12] Diaz R. J. Rosenberg R. (2008). Spreading dead zones and consequences for marine ecosystems. Science 321, 926–929. 10.1126/science.1156401 18703733

[B13] Egger M. Rasigraf O. Sapart C. J. Jilbert T. Jetten M. S. M. Röckmann T.. (2015). Iron-mediated anaerobic oxidation of methane in brackish coastal sediments. Environ. Sci. Technol. 49, 277–283. 10.1021/es503663z 25412274

[B14] Emeis K. –C. Neumann T. Endler R. Struck U. Kunzendorf H. Christiansen C. (1998). Geochemical records of sediments in the Eastern Gotland Basin – products of sediment dynamics in a not-so-stagnant anoxic basin? Appl. Geochem. 13, 349–358. 10.1016/S0883-2927(97)00104-2

[B15] Ferdelman T. G. Lee C. Pantoja S. Harder J. Bebout B. M. Fossing H. (1997). Sulfate reduction and methanogenesis in *Thioploca-*dominated sediment off the coast of Chile. Geochim. Cosmochim. Acta 61, 3065–3079. 10.1016/S0016-7037(97)00158-0

[B16] Gartman A. Yücel M. Madison A. S. Chu D. W. Ma S. Janzen C. P. (2011). Sulfide oxidation across diffuse flow zones of hydrothermal vents. Aquatic Geochem. 17, 583–601. 10.1007/s10498-011-9136-1

[B17] Glazer B. T. Cary S. C. Hohmann L. Luther G. W. III (2002). Sulfur speciation and microbial characterization of an intertidal salt marsh microbial mat, in Environmental Electrochemistry: Analysis of Trace Element Biogeochemistry, Vol. 811, American Chemical Society Symposium Series, eds Taillefert M. Rozan T. F. (Washington, DC), 283–304.

[B18] Grünke S. Felden J. Lichtschlag A. Girnth A.-C. De Beer D. Wenzhoefer F.. (2011). Niche differentiation among mat-forming, sulfide-oxidizing bacteria at cold seeps of the Nile Deep Sea Fan (Eastern Mediterranean Sea). Geobiology 9, 330–348. 10.1111/j.1472-4669.2011.00281.x 21535364

[B19] Hagen E. Feistel R. (2004). Observations of low-frequency current fluctuations in deep water of the Eastern Gotland Basin/Baltic Sea. J. Geophys. Res. 109, 1–15. 10.1029/2003jc002017

[B20] Hannson M. Andersson L. Axe P. Szaron J. (2013). Oxygen Survey in the Baltic Sea – Extent of Anoxia and Hypoxia, 1960-2012. Report Oceanography No. 46, Swedish Meteorological and Hydrological Institute, Goteborg.

[B21] Heiser U. Neumann T. Scholten J. Stuben D. (2001). Recycling of manganese from anoxic sediments in stagnant basins by seawater inflow: a study of surface sediments from the Gotland Basin, Baltic Sea. Mar. Geol. 177, 151–166. 10.1016/S0025-3227(01)00129-3

[B22] Hille S. Leipe T. Seifert T. (2006). Spatial variability of recent sedimentation rates in the Eastern Gotland Basin (Baltic Sea). Oceanologia 48, 297–317.

[B23] Holmkvist L. Kamyshny A. Brüchert V. Jørgensen B. B. (2014). Sulfidization of lacustrine glacial clay upon Holocene marine transgression (Arkona Basin, Baltic Sea). Geochim. Cosmochim. Acta 142, 75–94. 10.1016/j.gca.2014.07.030

[B24] Jessen G. L. Lichtschlag A. Struck U. Boetius A. (2016). Distribution and composition of thiotrophic mats in the hypoxic zone of the Black Sea (150-170 m water depth, Crimea margin). Front. Microbiol. 7:1011. 10.3389/fmicb.2016.01011 27446049 PMC4925705

[B25] Jilbert T. Slomp C. P. (2013a). Rapid high-amplitude variability in Baltic Sea hypoxia during the Holocene. Geology 41, 1182–1186. 10.1130/G34804.1

[B26] Jilbert T. Slomp C. P. (2013b). Iron and manganese shuttles control the formation of authigenic phosphorus minerals in the euxinic basins of the Baltic Sea. Geochim. Cosmochim. Acta 107, 155–169. 10.1016/j.gca.2013.01.005

[B27] Jonsson P. Carman R. Wulff F. (1990). Laminated sediments in the Baltic: a tool for evaluating nutrient mass balances. Ambio 19, 152–158.

[B28] Jørgensen B. B. Kasten S. (2006). Sulfur cycling and methane oxidation, in Marine Geochemistry, 2nd Edn., eds Schulz H. D. Zabel M. (Berlin: Springer), 271–309.

[B29] Jørgensen B. B. Nelson D. C. (2004). Sulfide oxidation in marine sediments: geochemistry meets microbiology, in Sulfur Biogeochemistry—Past and Present: Geological Society of America Special Paper, Vol. 379, eds Amend J. P. Edwards K. J. Lyons T. W. (Boulder, CO: Geological Society of America), 63–81.

[B30] Kamyshny A. Jr. Ferdelman T. G. (2010). Dynamics of zero-valent sulfur species including polysulfides at seep sites on intertidal sand flats (Wadden Sea, North Sea). Mar. Chem. 121, 17–26. 10.1016/j.marchem.2010.03.001

[B31] Kamyshny A. Jr. Goifman A. Gun J. Rizkov D. Lev O. (2004). Equilibrium distribution of polysulfide ions in aqueous solutions at 25°C: a new approach for the study of polysulfides equilibria. Environ. Sci. Technol. 38, 6633–6644. 10.1021/es049514e 15669322

[B32] Kamyshny A. Jr. Yakushev E. V. Jost G. Podymov O. I. (2013). Role of Sulfide Oxidation Intermediates in the Redox Balance of the Oxic-Anoxic Interface of the Gotland Deep, Baltic Sea, in Chemical Structure of Pelagic Redox Interfaces: Observations and Modeling, ed Yakushev E. V. (Berlin: Springer), 95–119.

[B33] Kamyshny A. Jr. Zilberbrand M. Ekeltchik I. Voitsekovski T. Gun J. Lev O. (2008). Speciation of polysulfides and zerovalent sulfur in sulfide-rich water wells in southern and central Israel. Aquat. Geochem. 14, 171–192. 10.1007/s10498-008-9031-6

[B34] Konovalov S. K. Luther G. W. Yücel M. (2007). Porewater redox processes and sediments in the Black Sea sediments. Chem. Geol. 245, 254–274. 10.1016/j.chemgeo.2007.08.010

[B35] Kühl M. Revsbech N. P. (2001). Biogeochemical microsensors for boundary layer studies, in The Benthic Boundary Layer: Transport Processes and Biogeochemistry, eds Boudreau B. P. Jørgensen B. B. (Oxford: Oxford University Press), 180–210.

[B36] Lavik G. Stührmann T. Brüchert V. Van der Plas A. Mohrholz V. Lam P.. (2009). Detoxification of sulphidic African shelf waters by blooming chemolithotrophs. Nature 457, 581–584. 10.1038/nature07588 19078958

[B37] Lenz C. Behrends T. Jilbert T. Silverira M. Slomp C. P. (2014). Redox-dependent changes in manganese speciation in Baltic Sea sediments from the Holocene Therman maximum: an EXAFS, XANES and LA-ICP-MC study. Chem. Geol. 370, 49–57. 10.1016/j.chemgeo.2014.01.013

[B38] Lichtschlag A. Kamyshny A. Jr. Ferdelman T. DeBeer D. (2013). Intermediate sulfur oxidation state compounds in the euxinic surface sediments of the Dvurechenskii mud volcano (Black Sea). Geochim. Cosmochim. Acta 105, 130–145. 10.1016/j.gca.2012.11.025

[B39] Luther G. W. Brendel P. J. Lewis B. L. Sundby B. Lefrancois L. Silverberg N. (1998). Simultaneous mueasurement of O2, Mn, Fe, I- and S(-II) in marine pore waters with a solid-state voltammetric microelectrode. Limnol. Oceanogr. 43, 325–333. 10.4319/lo.1998.43.2.0325

[B40] Luther G. W. Glazer B. T. Ma S. Trouwborst R. E. Moore T. S. Metzger E. (2008). Use of voltammetric solid-state (micro)electrodes for studying biogeochemical processes: laboratory measurements to real time measurements with an *in situ* electrochemical analyzer (ISEA). Mar. Chem. 108, 221–235. 10.1016/j.marchem.2007.03.002

[B41] Luther G. W. III Glazer B. T. Hohmann L. Popp J. I. Taillefert M. Rozan T. F.. (2001). Sulfur speciation monitored *in situ* with solid state gold amalgam voltammetric microelectrodes: polysulfides as a special case in sediments, microbial mats and hydrothermal vent waters. J. Environ. Monit. 3, 61–66. 10.1039/b006499h 11253020

[B42] Matthaeus W. (1995). Natural variability and human impacts reflected in long-term changes in the Baltic deep water conditions – A brief review. Deutsche Hydrographische Zeitschrift 47, 47–65. 10.1007/BF02731990

[B43] McGinnis D. F. Cherednichenko S. Sommer S. Berg P. Rovelli L. Schwarz R. (2011). Simple, robust eddy correlation amplifier for aquatic dissolved oxygen and hydrogen sulfide flux measurements. Limnol. Oceanogr. 9, 340–347. 10.4319/lom.2011.9.340

[B44] Meyer D. Prien R. D. Dellwig O. Waniek J. J. Schulz-Bull D. (2014). Electrode measurements of the oxidation reduction potential in the Gotland Deep using a moored profiling instrumentation. Estuarine Coast. Shelf Sci. 141, 26–36. 10.1016/j.ecss.2014.02.001

[B45] Mohrholz V. Naumann M. Nausch G. Krüger S. Graewe U. (2015). Fresh oxygen for the Baltic Sea – An exceptional saline inflow after a decade of stagnation. J. Mar. Syst. 148, 152–166. 10.1016/j.jmarsys.2015.03.005

[B46] Neretin L. N. Pohl C. Jost G. Leipe T. Pollehne F. (2003). Manganese cycling in the Gotland Deep, Baltic Sea. Mar. Chem. 82, 125–143. 10.1016/S0304-4203(03)00048-3

[B47] Noffke A. Sommer S. Dale A. W. Hall P. O. J. Pfannkuche O. (2016). Benthic nutrient fluxes in the Eastern Gotland Basin (Baltic Sea) with particular focus on microbial mat ecosystems. J. Mar. Syst. 158, 1–12. 10.1016/j.jmarsys.2016.01.007

[B48] Pasteris J. D. Freeman J. D. Goffredi S. K. Buck K. R. (2001). Raman spectroscopic and laser scanning confocal microscopic analysis of sulfur in living sulfur-precipitating marine bacteria. Chem. Geol. 180, 3–18. 10.1016/S0009-2541(01)00302-3

[B49] Pfeffer C. Larsen S. Song J. Dong M. Besenbacher F. Meyer R. L.. (2012). Filamentous bacteria transport electrons over centimetre distances. Nature 491, 218–221. 10.1038/nature11586 23103872

[B50] Poulton S. W. Krom M. D. Raiswell R. (2004). A revised scheme for the reactivity of iron (oxyhydr)oxide minerals towards dissolved sulfide. Geochim. Cosmochim. Acta 68, 3703–3715. 10.1016/j.gca.2004.03.012

[B51] Preisler A. de Beer D. Lichtschlag A. Lavik G. Boetius A. Jørgensen B. B. (2007). Biological and chemical sulfide oxidation in a Beggiatoa inhabited marine sediment. ISME J. 2007, 341–353. 10.1038/ismej.2007.50 18043645

[B52] Revsbech N. P. Jørgensen B. B. (1983). Photosynthesis of benthic microflora measured with high spatial resolution by the oxygen microprofile method: capabilities and limitations of the method. Limnol. Oceanogr. 28, 749–756. 10.4319/lo.1983.28.4.0749

[B53] Rickard D. Luther G. W. (2007). Chemistry of iron sulfides. Chem. Rev. 107, 514–562. 10.1021/cr0503658 17261073

[B54] Rozan T. F. Theberge S. M. Luther G.W. III (2000). Quantifying elemental sulfur (S0), bisulfide (HS-) and polysulfides (Sx2-) using a voltammetric method. Anal. Chim. Acta 415, 175–184. 10.1016/S0003-2670(00)00844-8

[B55] Scholz F. McManus J. Sommer S. (2013). The manganese and iron shuttle in a modern euxinic basin and implications for molybdenum cycling at euxinic ocean margins. Chem. Geol. 355, 56–68. 10.1016/j.chemgeo.2013.07.006

[B56] Schulz H. N. Jørgensen B. B. (2001). Big Bacteria. Annu. Rev. Microbiol. 55, 105–137. 10.1146/annurev.micro.55.1.105 11544351

[B57] Schunck H. Lavik G. Desai D. K. Großkopf T. Kalvelage T. Löscher C. R.. (2013). Giant hydrogen sulfide plume in the oxygen minimum zone off peru supports chemolithoautotrophy. PLoS ONE 8:e68661. 10.1371/journal.pone.0068661 23990875 PMC3749208

[B58] Schwedt A. Kreutzmann A.-C. Polerecky L. Schulz-Vogt H. N. (2012). Sulfur respiration in a marine chemolithotrophic Beggiatoa strain. Front. Microbiol. 2:276. 10.3389/fmicb.2011.00276 22291687 PMC3253548

[B59] Seitaj D. Schauer R. Sulu-Gambari F. Hidalgo-Martinez S. Malkin S. Y. Burdorf L. D. W.. (2015). Cable bacteria generate a firewall against euxinia in seasonally hypoxic basins. Proc. Natl. Acad. Sci. U.S.A. 112, 13278–13283. 10.1073/pnas.1510152112 26446670 PMC4629370

[B60] Slowey A. J. Marvin-Dipasquale M. (2012). How to overcome inter-electrode variability and instability to quantify dissolved oxygen, Fe(II), mn(II), and S(-II) in undisturbed soils and sediments using voltammetry. Geochem. Trans. 13:6. 10.1186/1467-4866-13-6 22731822 PMC3442984

[B61] Soetaert K. Petzoldt T. Meysman F. (2012). Marelac: Tools For Aquatic Sciences. R package version 2.1.2. Available online at: http://CRAN.R-project.org/package=marelac

[B62] Sohlenius G. Sternbeck J. Andren E. Westman P. (1996). Holocene history of the Baltic Sea as recorded in a sediment core from the Gotland Deep. Mar. Geol. 134, 183–201. 10.1016/0025-3227(96)00047-3

[B63] Sommer S. Clemens D. Yücel M. Pfannkuche O. Hall P. Almroth Rosell E. (2017). Major bottom water ventilation events do not significantly reduce basin-wide benthic N and P release in the Eastern Gotland Basin (Baltic Sea). Front. Mar. Sci. 4:18 10.3389/fmars.2017.00018

[B64] Sommer S. Gier J. Treude T. Lomnitz U. Dengler M. Cardich J. (2016). Depletion of oxygen, nitrate and nitrite in the Peruvian oxygen minimum zone cause an imbalance of benthic nitrogen fluxes. Deep-Sea Res. I 112, 113–122. 10.1016/j.dsr.2016.03.001

[B65] Sommer S. Linke P. Pfannkuche O. Niemann H. Treude T. (2010). Benthic respiration in a seep habitat dominated by dense beds of ampharetid polychaetes at the Hikurangi Margin (New Zealand). Mar. Geol. 272, 223–232. 10.1016/j.margeo.2009.06.003

[B66] Sternbeck J. Sohlenius G. (1997). Authigenic sulfide and carbonate mineral formation n Holocene sediments of the Baltic Sea. Chem. Geol. 135, 55–73. 10.1016/S0009-2541(96)00104-0

[B67] Sun M. Mu Z.-X. Chen Y.-P. Sheng G.-P. Liu X.-W. Chen Y.-Z.. (2009). Microbe-assisted sulfide oxidation in the anode of a microbial fuel cell. Environ. Sci. Technol. 43, 3372–3377. 10.1021/es802809m 19534160

[B68] Taillefert M. Luther G. W. III Nuzzio D. B. (2000). The application of electrochemical tools for *in situ* measurements in aquatic systems. Electroanalysis 12, 401–412. 10.1002/(SICI)1521-4109(20000401)12:6<401::AID-ELAN401>3.0.CO;2-U

[B69] Taillefert M. Neuhuber S. Bristow G. (2007). The effect of tidal forcing on biogeochemical processes in intertidal salt marsh sediments. Geochem. Trans. 8:6. 10.1186/1467-4866-8-6 17567893 PMC1904194

[B70] Theberge S. M. Luther G. W. (1997). Determination of the electrochemical properties of a soluble aqueous FeS cluster present in sulfidic systems. Aquat. Geochem. 3, 191–211. 10.1023/A:1009648026806

[B71] Vallius H. Kunzendorf H. (2001). Sediment surface geochemistry of three Baltic Sea deep basins. AMBIO 30, 135–141. 10.1579/0044-7447-30.3.135 11436660

[B72] Wenzhöfer F. Holby O. Glud R. N. Nielsen H. K. Gundersen J. K. (2000). *In situ* microsensor studies of a shallow water hydrothermal vent at Milos, Greece. Mar. Chem. 69, 43–54. 10.1016/S0304-4203(99)00091-2

[B73] Winogradow A. Pempkowiak J. (2014). Organic carbon burial rates in the Baltic Sea sediments. Estuarine Coast. Shelf Sci. 138, 27–36. 10.1016/j.ecss.2013.12.001

[B74] Yücel M. (2013). Down the thermodynamic ladder: a comparative study of marine redox gradients across diverse sedimentary environments. Estuarine Coast. Shelf Sci. 131, 83–92. 10.1016/j.ecss.2013.07.013

[B75] Yücel M. Luther G. W. Moore W. S. (2010). Earthquake-induced turbidite deposition as a previously unrecognized sink for hydrogen sulfide in the Black Sea sediments. Mar. Chem. 121, 176–186. 10.1016/j.marchem.2010.04.006

[B76] Zillen L. Conley D. J. Andren T. Andren E. Bjorck S. (2008). Past occurrences of hypoxia in the Baltic Sea and the role of climate variability, environmental change and human impact. Earth-Sci. Rev. 91, 77–92. 10.1016/j.earscirev.2008.10.001

[B77] Zopfi J. Böttcher M. E. Jørgensen B. B. (2008). Biogeochemistry of sulfur and iron in *Thioploca*-colonized surface sediments in the upwelling area off central Chile. Geochim. Cosmochim. Acta 72, 827–843. 10.1016/j.gca.2007.11.031

