# Supplementary figures and images for: Microbial Sulfide Filter along a Benthic Redox Gradient in the Eastern Gotland Basin, Baltic Sea
Source: Front Microbiol. 2017 Feb 9;8:169. doi: 10.3389/fmicb.2017.00169 (PMC5299003; doi:10.3389/fmicb.2017.00169)

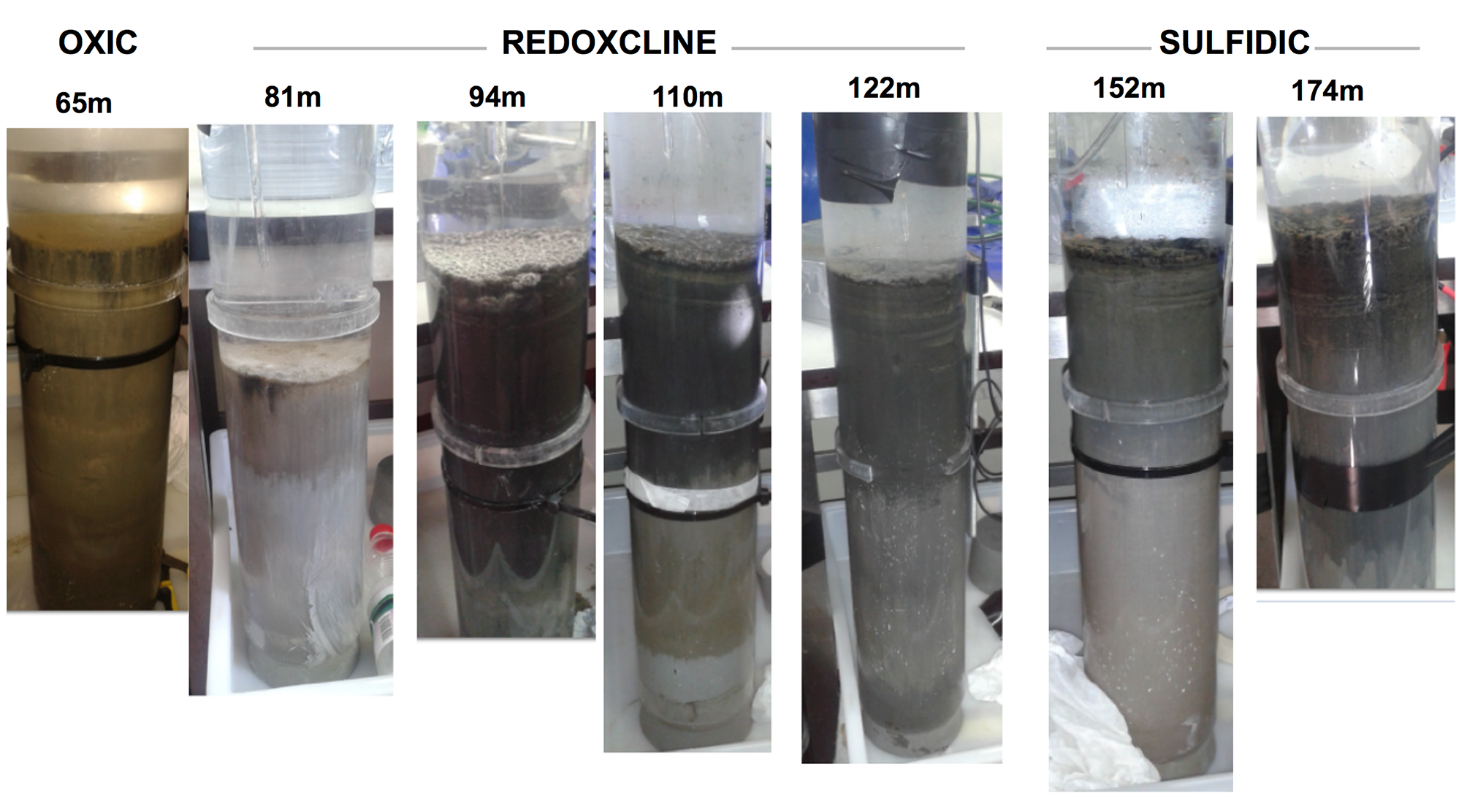

Supplement: Figure S1 — Photographs of the MUC cores taken from the transect. [file Image1.TIFF]

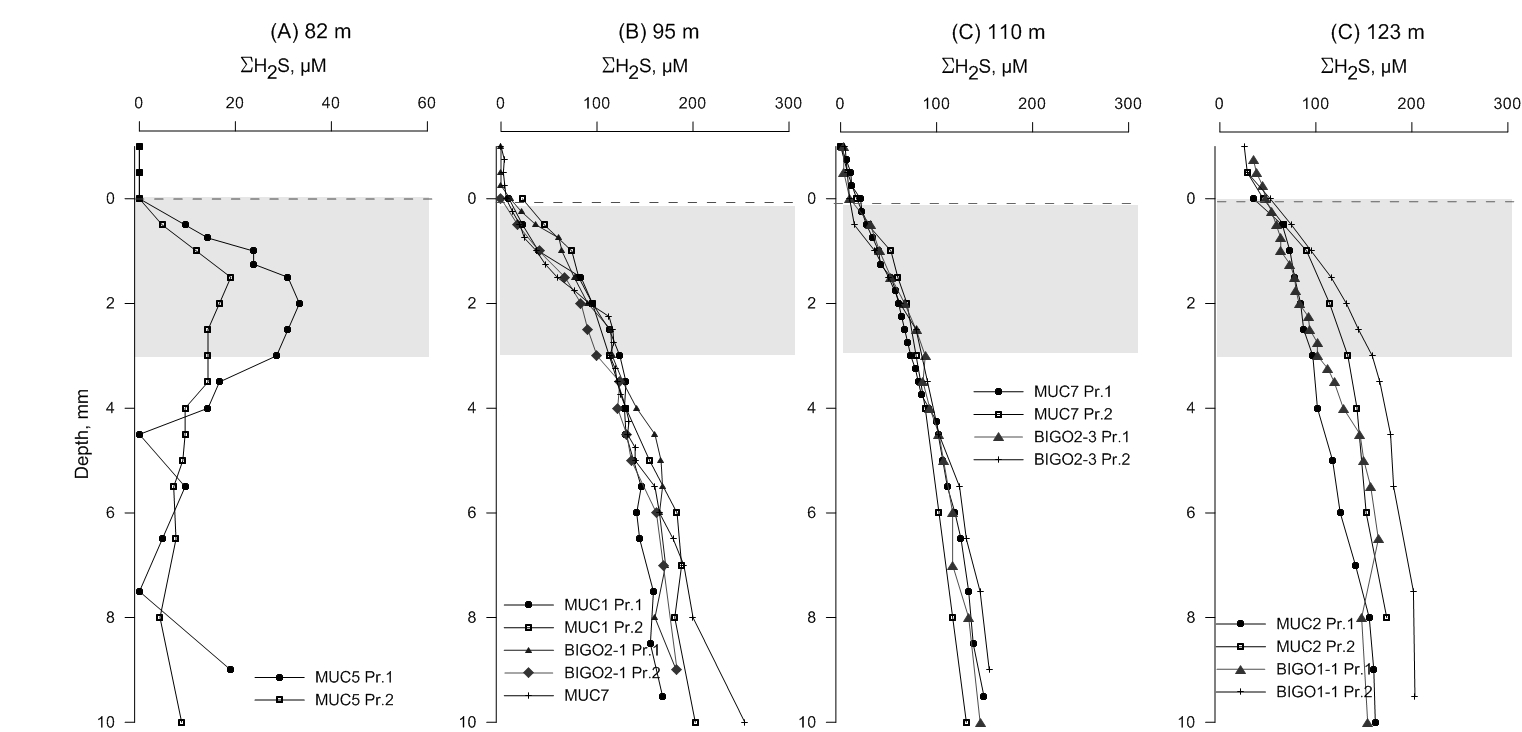

Supplement: Figure S2 — Zoomed (top 10 mm) version of the graphs in Figure 4. [file Image2.JPEG]

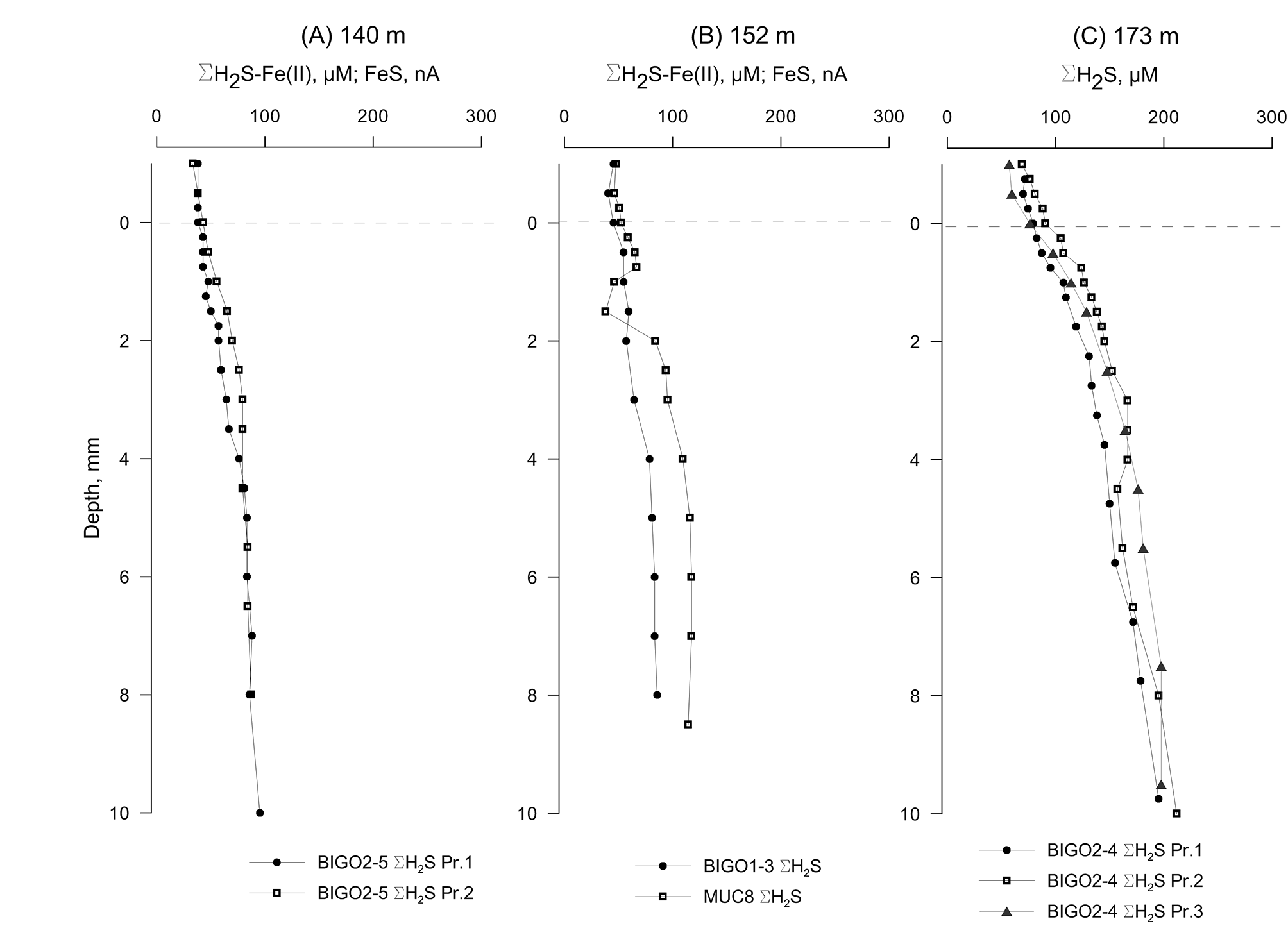

Supplement: Figure S3 — Zoomed (top 10 mm) version of the graphs in Figure 6. [file Image3.TIF]

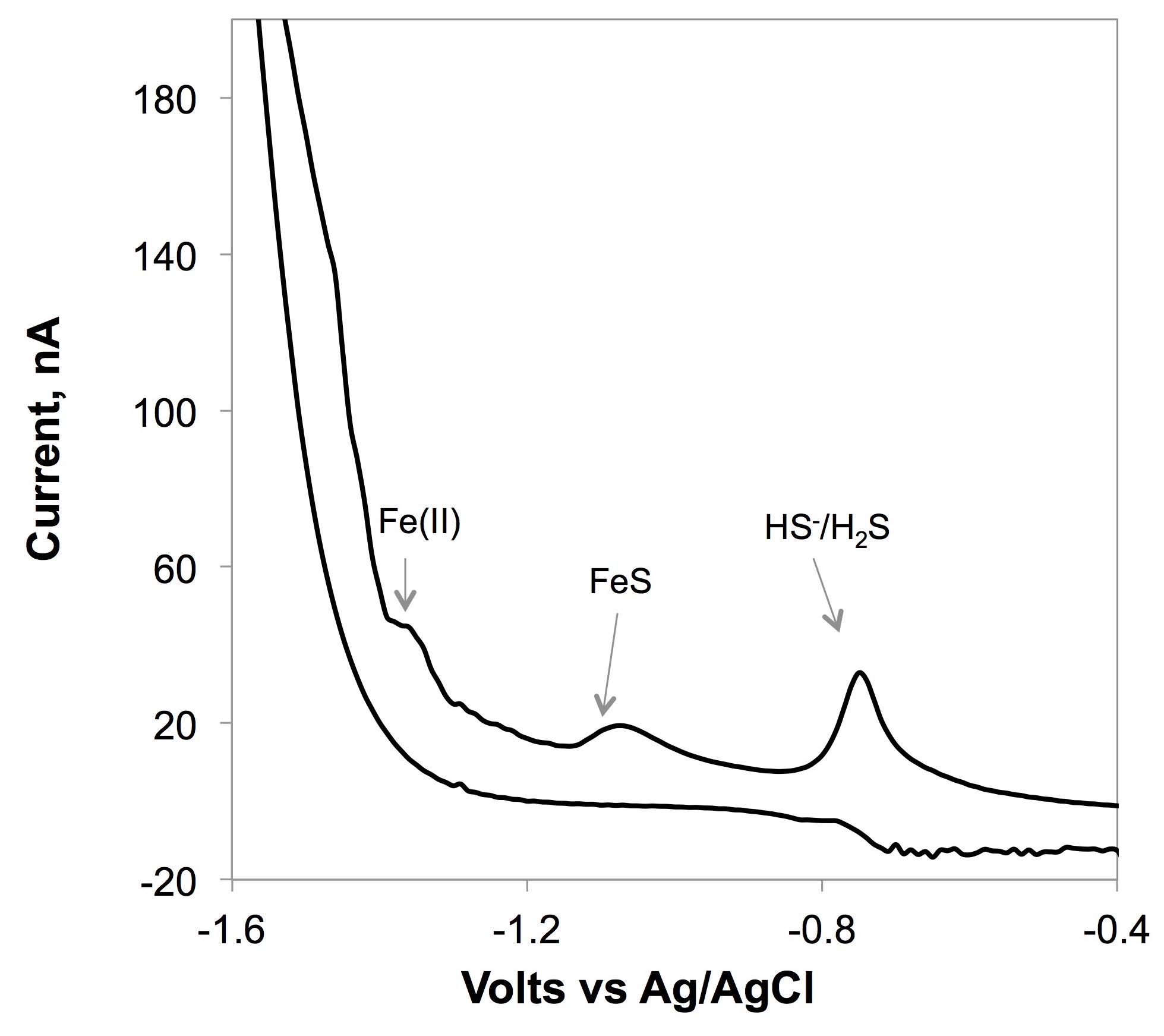

Supplement: Figure S4 — Representative voltammetric scan showing the coexistence of Fe(II) and FeS (from the 152 m-core, 100 mm). [file Image4.JPEG]
